# Latest Research Progress in High-Purity Material Purification Technology

**DOI:** 10.1021/acsomega.5c03602

**Published:** 2025-08-13

**Authors:** Pan Zhao, Jingwen Qiu, Hongjia Liang, Yijing Jiang, Fangdi Huang, Nannan Wang, Yanqiu Zhu

**Affiliations:** † State Key Laboratory of Featured Metal Materials and Life-Cycle Safety for Composite Structures, MOE Key Laboratory of New Processing Technology for Nonferrous Metals and Materials, School of Resources, Environment and Materials, 12664 Guangxi University, Nanning 530004, China; ‡ Faculty of Environment, Science and Economy, 3286 University of Exeter, Exeter EX4 4QF, U.K.

## Abstract

High-purity metals, defined as metals with impurity levels minimized to achieve purity, typically ≥99.999% (5N grade), constitute critical raw materials and serve as essential supporting components for modern high-technology industries. Common examples include high-purity indium, gallium, germanium, magnesium, lithium, aluminum, tin, tellurium, and titanium. These materials find extensive applications in semiconductor manufacturing, aerospace engineering, energy technologies, and healthcare sectors. The exceptionally low impurity content confers superior properties upon high-purity metals compared to those of their industrial-grade counterparts. Consequently, products fabricated from these materials exhibit enhanced performance, stability, and controllability, thereby meeting the stringent requirements of downstream high-precision applications. This review comprehensively examines established techniques for the preparation and purification of high-purity metals, encompassing extraction, ion exchange, electrolysis, zone melting, distillation, and single-crystal growth, and summarizes the state-of-the-art advancements in these methodologies.

## Graphical abstract

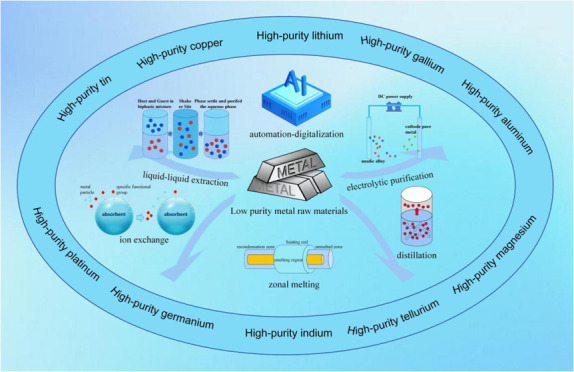

## 1 Introduction

High-purity metals are key raw materials for industry and are known as important support materials for modern high-tech industries. High-purity metals, i.e., metals with high-purity and low-impurity contents, are usually required to have a purity of 5N grade (99.999%) or higher. The use of various purification and preparation methods to improve the purity of the metal, which can better utilize the metal properties of high-purity metals, is an important research direction in the field of metals. Common high-purity metals include high-purity indium, high-purity gallium, high-purity germanium, high-purity magnesium, high-purity lithium, high-purity aluminum, high-purity tin, high-purity tellurium, and high-purity titanium. Table 1 lists the physical and chemical properties and application scenarios of common high-purity metals.

**Table 1:** Physical and Chemical Properties and Application Scenarios of Common High-Purity Metals

high-purity metal	physical and chemical properties	application scenarios
indium^1–3^	low melting point, high boiling point, high ductility, high conductivity	ITO thin-film transistors, photovoltaic copper indium gallium selenide (CIGS), pharmaceutical compounds, high-performance infrared detectors, light-emitting diodes (LEDs), etc
gallium^4,5^	excellent photoelectric properties, thermal stability, low melting point, high boiling point	semiconductors, light-emitting diodes (LEDs), photodetectors, solar cells, and medical devices
germanium^6,7^	high carrier mobility, high dielectric constant, high melting point, high boiling point	infrared optics, fiber optics, catalysts, semiconductors, anode materials, solar energy applications and medical, etc
magnesium^8,9^	low density, high melting point, high boiling point, good electrical and thermal conductivity, biocompatibility	aerospace, automotive, 3C (computers, communications, and consumer electronics), biomedical and energy, etc
lithium^10,11^	low density, good electrochemical properties, good thermal conductivity, high reflectivity	functional ceramics, high-performance glass industry, metallurgy, aerospace, nuclear energy, pharmaceuticals, and energy storage
aluminum^12–14^	low density, lightweight, high specific strength, excellent thermal conductivity, mechanical properties, and corrosion resistance	automobiles, airplanes, military weapons, etc
tin^15,16^	high stability, high conductivity, and excellent solderability	gas sensing, transparent conductive electrodes and liquid crystal displays, etc
tellurium^17–19^	good plasticity and ductility, semiconductor properties	gas sensors, phase-change memory chips, photovoltaic modules, thermoelectric devices, etc
titanium^20,21^	excellent corrosion resistance, low density, and high strength	aerospace, chemical, defense, military, etc

With the continuous development of high technology, the demand for high-purity metal and the purity requirements is increasing; impurities in high-purity metal will affect the performance of the metal, the higher the purity of high-purity metal, high-purity metal synthesized alloys or devices, the better the performance of the device.^22,23^ In the case of infrared detectors made of indium, for example, the performance of synthesized indium compounds is better and more stable and controllable as the purity of indium increases, and the purity of indium is usually required to be 6N or even 7N or more, which leads to better infrared detector performance.^24^

With the downstream high-precision field of high-purity metal purity requirements continuing to improve, we need to continue to innovate and improve the purification of high-purity metal preparation technology; so, this paper shows a comprehensive review on the current purification of a variety of high-purity metal purification technology.

## 2 Purification and Preparation Methods

At present, the purification of high-purity metal preparation methods mainly includes extraction, ion exchange, electrolysis, regional melting method, distillation, single-crystal pulling method, and adsorption method. Different purification preparation methods have different characteristics and advantages and disadvantages and require different physical and chemical properties of different metals using appropriate purification methods and can be used jointly by a variety of purification methods, so that the metal achieves higher purity to meet the downstream high-tech field of high-purity metal purity requirements.

### 2.1 Ion-Exchange Method

Ion exchange typically separates target metals and impurities from the solution. The extraction of target metals is usually carried out using resins and has been utilized for the purification of indium, gallium, tin, germanium, lithium, and other metals. The use of different ions on different metal ions has a different binding effect principle to achieve the purpose of extraction and separation. Various types of ion-exchange membranes and ion-exchange resins work on the principle of ion-exchange method as shown in Figure 1.

**Figure 1:**
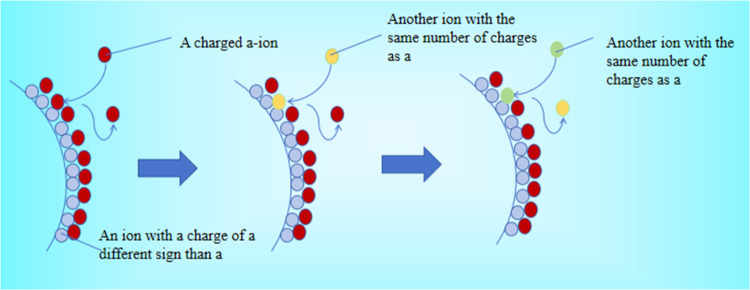
Principles of the ion-exchange method.

The ion-exchange method of the resin solid phase is commercially promising, and a number of studies have been carried out in the direction of resin modification. At their core, they all utilize the characteristics of a strong affinity between metal ions and exchangeable ions of the resin and a poor affinity between exchangeable ions and other impurity ions. In the extraction of indium by ion exchange, Adhikari et al. in 2012 proposed the use of methylene cross-linked calix[4]-arene and calix[6]-arene carboxylic acid resins for the extraction of indium, with the mechanism of action being the aggregation of the polyfunctional groups in the cuproaromatic hydrocarbons in the resins and the adsorption of the indium. The maximum adsorption of indium was found to be 109 mg/g of resin for calix[4]-arene resin and 213 mg/g for calix[6]-arene resin. These two resins showed a high uptake of indium and superior selective adsorption performance.^25^ In 2016, Lee et al. proposed the use of LewatitTP207 resin, which has 99% adsorption capacity and desorption efficiency for indium ions.^26^ In 2018, Assefi et al. used LewatitTP208, LewatitTP260, and AmberliteIRA743 resins, which recovered indium with 99% efficiency. These four resins all exhibit excellent selectivity for indium ions.^27^

The resins used for specific ion-exchange methods to extract different metals such as gallium, germanium, lithium, tin, and platinum vary. The ion-exchange method is also widely used for the extraction of gallium, and a polyacrylate-divinylbenzene isohydroxamic acid resin was proposed for the purification of gallium by Li et al. in 2024. By introducing the isohydroxamic acid group to chelate gallium preferentially by the electrostatic interaction, this resin has the highest adsorption efficiency of up to 97.75%, and the maximum adsorption amounts to 30.08 mg/g, which is a kind of resin with high selectivity for gallium.^28^ The introduction of some groups, functional groups, and the target metal ion adsorption, chelation, etc., to increase the attraction of the target metal ions to achieve the purpose of metal extraction is a conventional strengthening of the ion-exchange method of purification of metal ideas. In 2023, Raj et al. proposed a catechol derivative chemically modified polymer resin for gallium extraction, and the adsorption efficiency of the resin for gallium reached more than 60% after four adsorption–desorption cycles, demonstrating good reusability of the resin.^29^ In 2024, Qin et al. proposed a new type of the amidoxime-grafted polyacrylonitrile-styrene-divinylbenzene (A-PSD) resin for the extraction of gallium, which has an aluminum ion removal rate of more than 99% and a gallium ion desorption rate of 91.54%, reflecting the advantages of its good selectivity for gallium and a high rate of impurity removal.^30^

Similarly, the ion-exchange method can be used for the extraction of the metals germanium, lithium, tin, and platinum. In 2018, Cruz et al. discussed catechol-based resins, which can be stripped of more than 80% of all germanium extracted and which have excellent effective desorption.^31^ In 2024, He et al. proposed the D201×7 resin, which was found to have a germanium adsorption efficiency of over 97% and a germanium desorption efficiency of 95% after five adsorption–desorption cycles.^32^ In 2019, Arroyo et al. discussed the use of LewatitK2629 resin to extract lithium from seawater, which has a maximum desorption rate of up to 80% and performs desalination of seawater while extracting lithium, a technology that is currently immature but has a high potential for the development of its economic benefits.^33^ In addition, Marinho et al. investigated a strongly basic anion-exchange resin (Cl-form) for the simultaneous recovery of platinum, tin, and indium from specific spent catalysts by using different sequences of ion exchange and elution and found that the resin desorbed more than 99% of all of the metals and recovered more than 98%.^34^ In 2023, Alshebli et al. using LewatitK2629 resin under the EED (electro-electrodialysis) process can be used in the extraction of lithium metal and also has the ability to produce hydrogen, extraction of boron, the use of ion-exchange resins for boron and lithium recovery as well as the production of hydrogen electrodialysis process as shown in Figure 2.^35^

**Figure 2:**
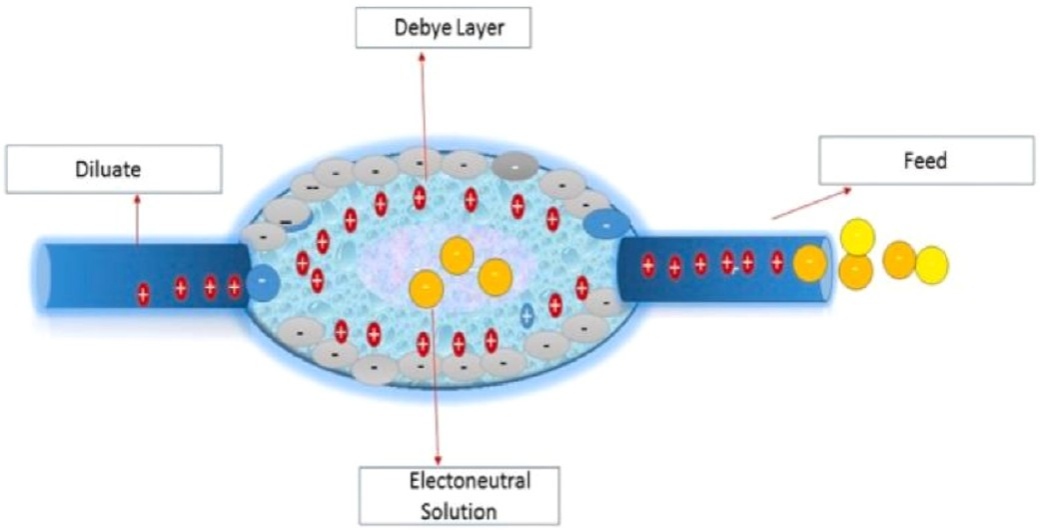
Transport of ions through ion-exchange membranes. Copyright 2023 The Authors. Published by Elsevier Ltd.

IEX-R (ion-exchange resin) combined with the EED process of the lithium and boron ion mass distribution, and removal rates are shown in Figure 3.^35^

**Figure 3:**
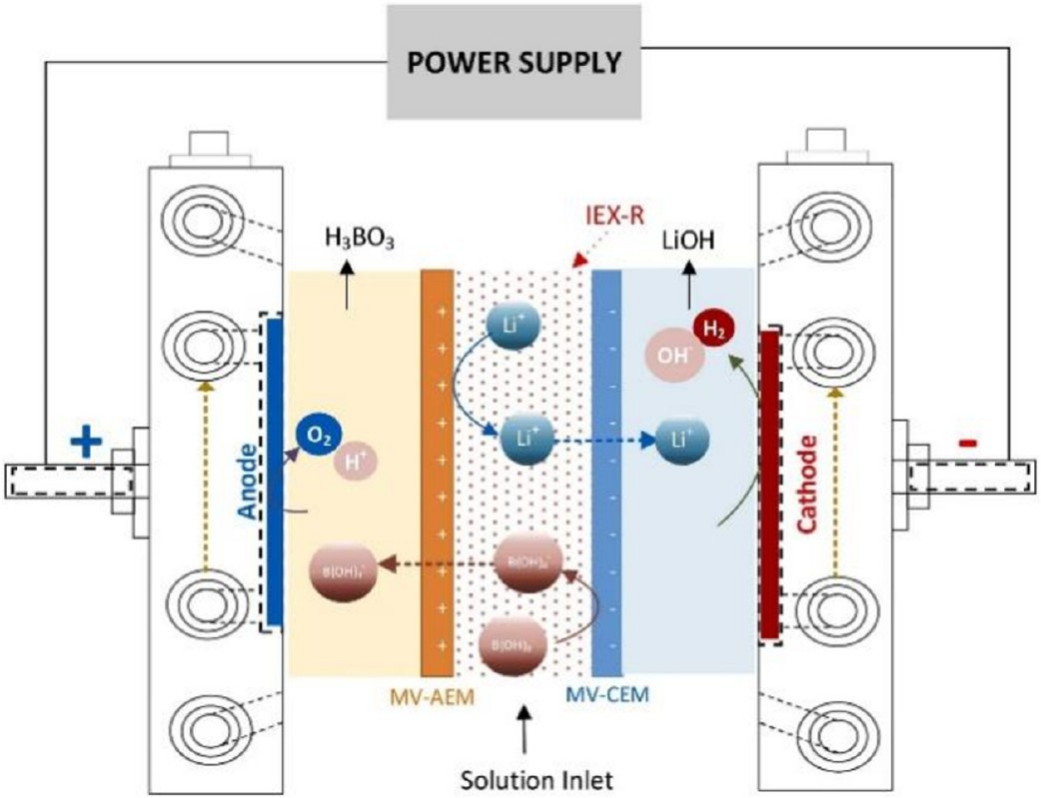
Schematic representation of the electro-electrodialysis process with ion-exchange resins for boron and lithium recovery and hydrogen production. Copyright 2023 The Author(s). Published by Elsevier Ltd.

It is found that a combination of IEX-R and EED process is more energy-saving and environmentally friendly than the previous process, and the energy-saving effect of the process is shown in Figure 4.^35^

**Figure 4:**
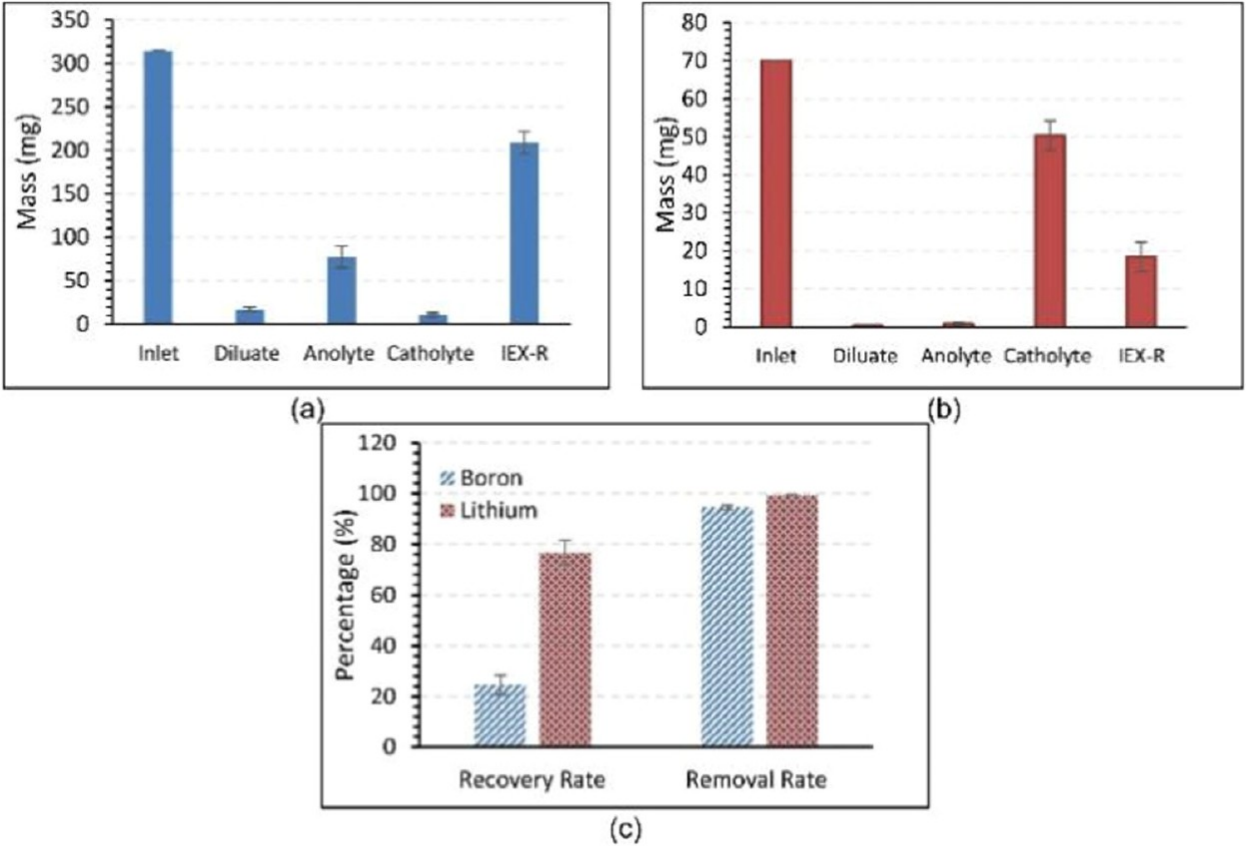
Measured ion mass in the reactor for experiments conducted by the EED process with IEX-R at an applied current density of 10 mA/cm^2^: (a) boron, (b) lithium, and (c) removal rates. Copyright 2023 The Author(s). Published by Elsevier Ltd.

The process can also produce hydrogen, boron, and other metals in the extraction of lithium metal.

In the ion-exchange method of purification of metals using adsorbent materials in addition to resins, there are inorganic ion-exchange absorbent, carbon-based ion-exchange absorbent, and ion-exchange membrane. Among them, the ion-exchange membrane relies on the membrane’s selectivity for ions, allowing only the movement of specific ions, thus achieving the removal and purification of ions. A schematic diagram of the anion-exchange membrane is shown in Figure 5.^36^

**Figure 5:**
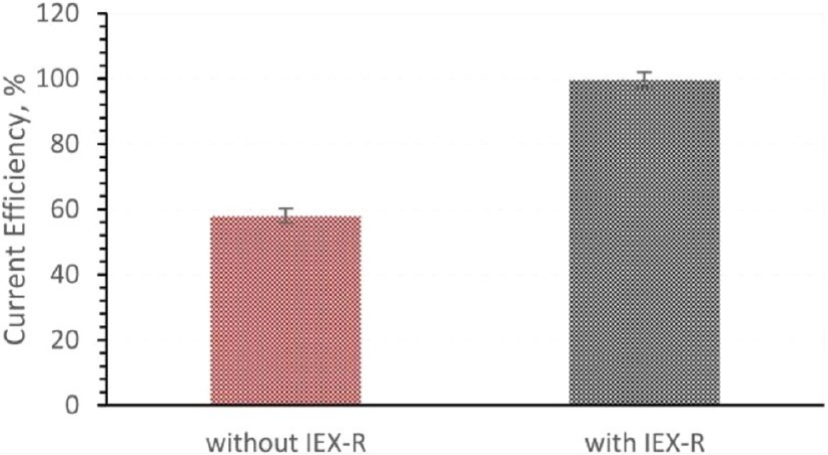
Current efficiencies of the EED process with and without IEX-R. Copyright 2023 The Author(s). Published by Elsevier Ltd.

In 2024, Peng et al. proposed a new zirconium-based adsorbent, Ma-Zr-MOF, as an efficient adsorbent for germanium recovery. The maximum adsorption of germanium by Ma-Zr-MOF was found to be 82.06 mg/g, and the adsorption rate of germanium by this resin could be maintained at more than 65% after 5 adsorption–desorption cycles.^37^ The researchers studied the improvement of the effectiveness of the ion-exchange method and also discussed the ion-exchangeable nanobeads, impregnating resin solvents, and ion-exchange process aspects. Kwak et al. discussed the ion-exchangeable nanobeads in the ion-exchange method; it was found that the maximum adsorption capacity of the nanobeads for indium was 0.78 mmol/g, and the nanobeads could also reach more than 0.65 mmol/g of indium after 10 adsorption–desorption cycles. This scheme is very promising in the field of indium extraction by ion exchange that reduces costs and is more environmentally friendly, saving pharmaceuticals.^38^

In 2024, Zhu et al. proposed weak acid leaching to extract gallium from red mud and found that the leaching rate of gallium could reach 95.9% after using phosphoric acid, and the leaching operation used phosphoric acid instead of sulfuric acid to dissolve gallium more fully into the solution for subsequent purification. This is a more environmentally friendly, green, low-cost processing method.^39^ Currently, the common solvents for impregnating resins used in the ion-exchange method are organophosphorus extractants, such as P507, D2EHPA, TBP, and TOPO, amine extractants, and ionic liquid extractants. In 2016, Wei et al. proposed a method of impregnating Cyanex 923 with HZ830 resin to extract isolated indium. The solvent-impregnated resin (SIR) prepared by SIR was found to be effective and stable in the adsorption and extraction of In. It was found that the adsorption rate could be more than 90%, and the adsorption and elution curves of the resin for indium were found to almost overlap after 5 adsorption–desorption cycles, which indicated that the regeneration performance of this resin was high.^40^ In response to the improved effectiveness of conventional ion-exchange removal, in 2022, Illés et al. proposed a selective leaching and complex anion-exchange process, which can produce pure indium from used liquid crystal display panels. This experiment also discusses that HCl has an effect on ion exchange, as shown in Figure 6.^41^

**Figure 6:**
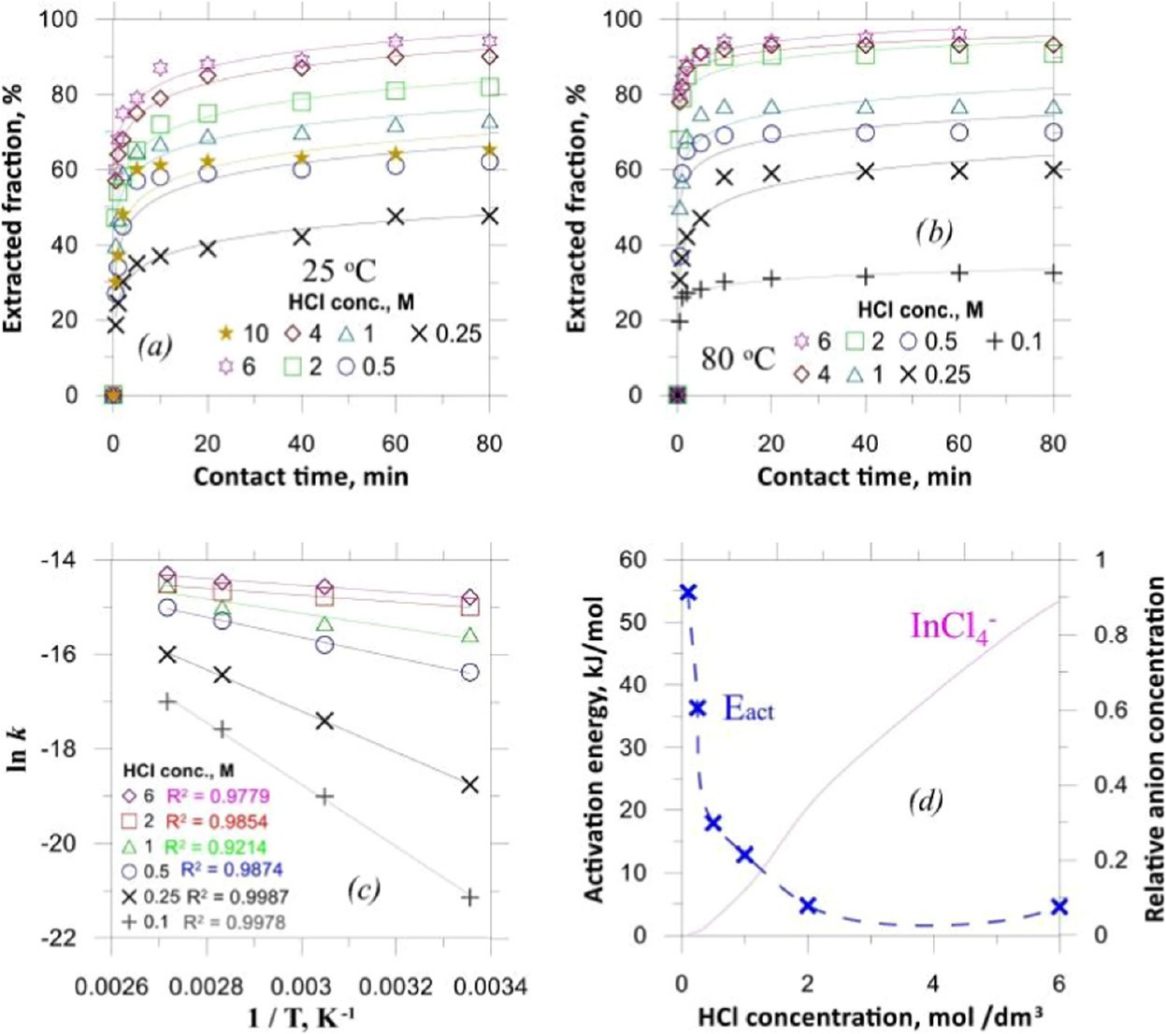
Anion-exchange kinetics of indium from 25 °C (a) and 80 °C (b) HCl solutions, the relevant Arrhenius plots (c), and the corresponding activation energies (d). Copyright 2022 The Author(s). Published by Elsevier Ltd.

The process is mainly achieved by adjusting the concentration of chlorine ions to control the adsorption of ionic species to achieve the separation of indium after the elimination of almost all impurities and then using the HCl solution to rinse the resin bed after the NaOH solution to remove impurities again. The core of this process is the concentration of chloride ions; the ionic form of indium chloride and sulfate at room temperature is shown in Figure 7.^41^

**Figure 7:**
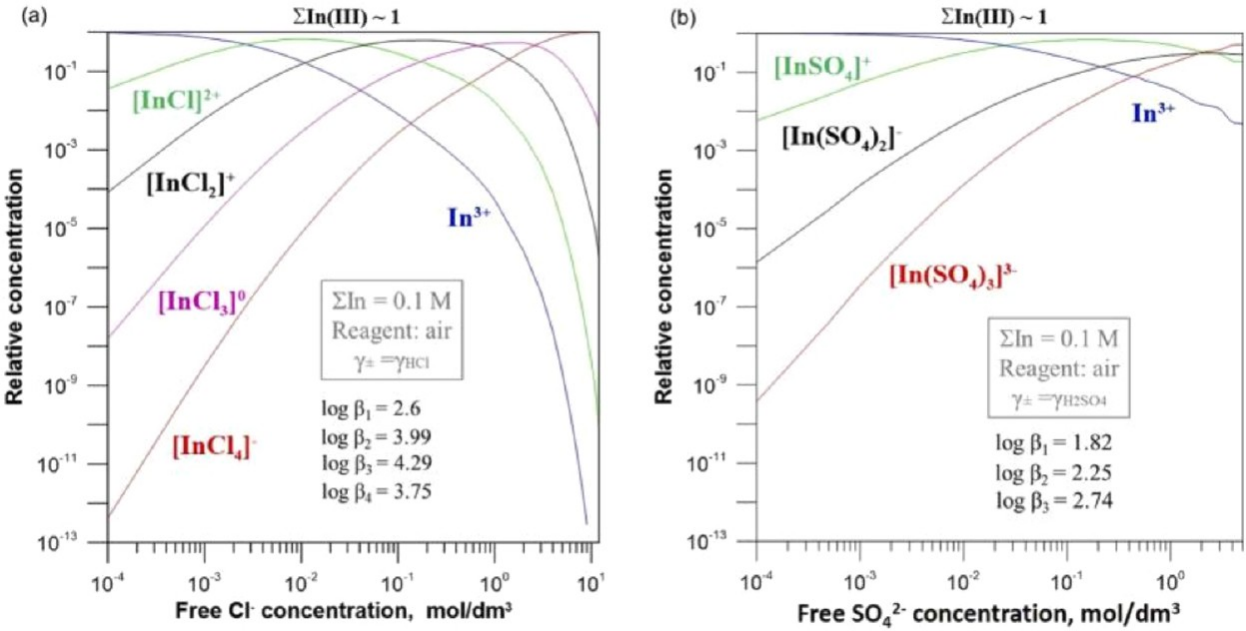
Ionic speciation of indium in chloride (a) and sulfate (b) solutions at 25 °C. Copyright 2022 The Author(s). Published by Elsevier Ltd.

And the effect of chloride ions on ion exchange is shown in Figure 8.^41^

**Figure 8:**
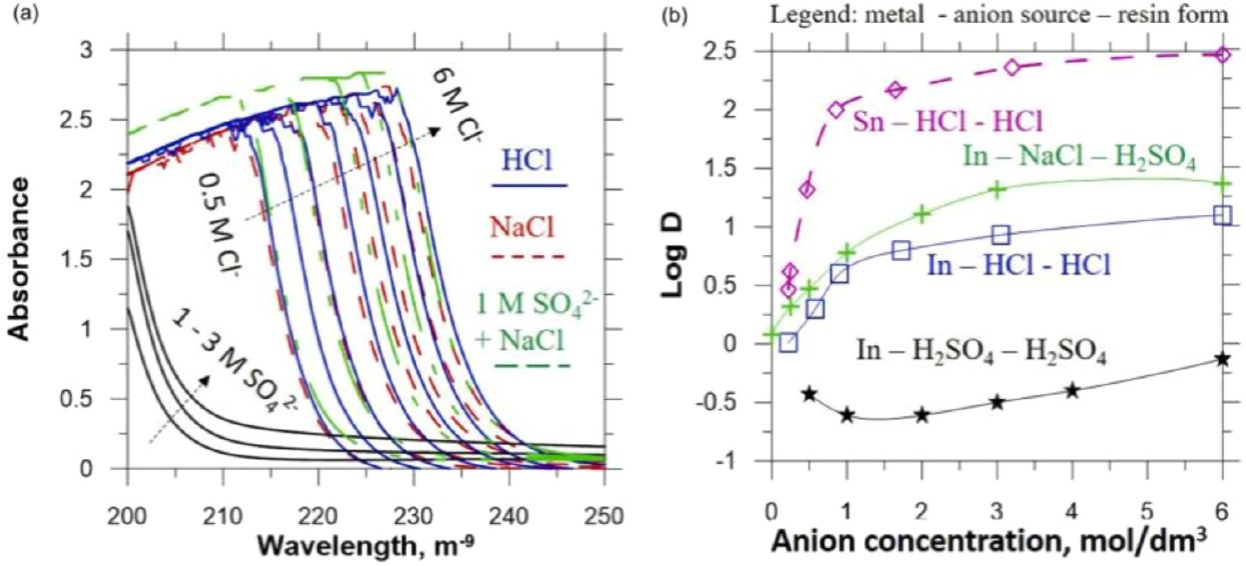
Effects of chloride ions on the absorption spectra of In­(III) (a) and the anion-exchange distribution functions (b) at 25 °C. Copyright 2022 The Author(s). Published by Elsevier Ltd.

After research and testing, the purity of this indium product can reach 99.9997% and the recovery rate of indium can be more than 90%.^41^

### 2.2 Extraction Method

The extraction method has been widely used in the field of metal purification; the extraction method is divided into liquid–liquid extraction and solid-phase extraction (SPE). The liquid–liquid extraction process diagram and a conceptual sketch are shown in Figure 9.^42^

**Figure 9:**
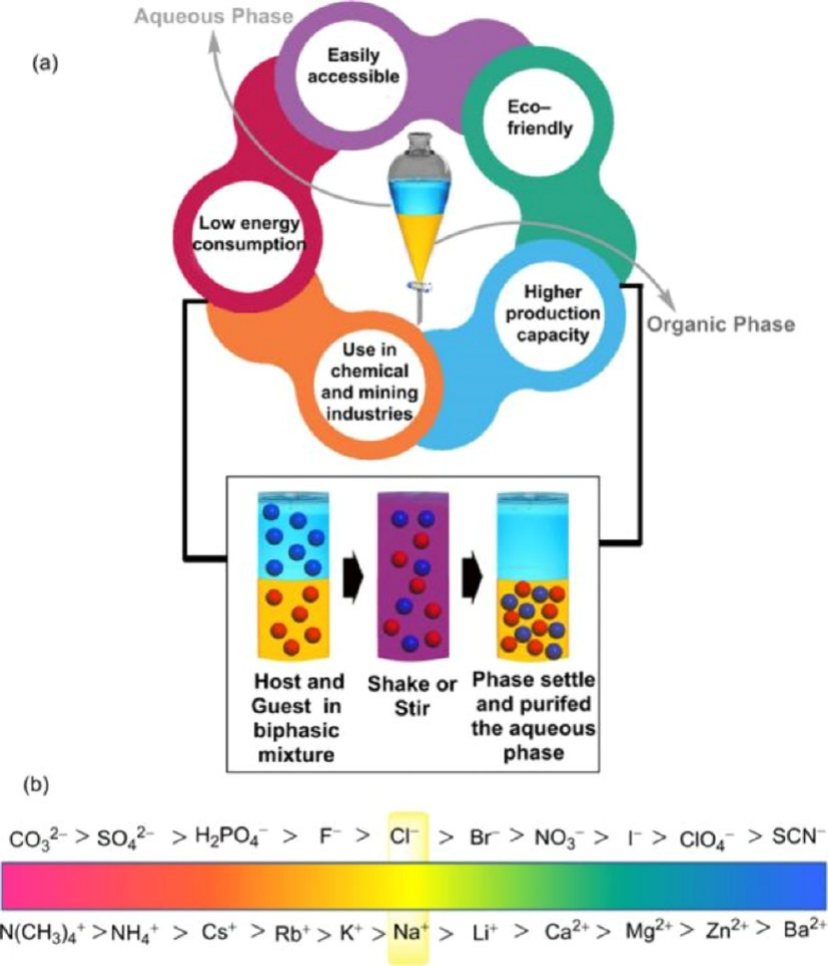
(a) Schematic illustration of the liquid–liquid extraction process. (b) Conceptual sketch of the Hofmeister series. Copyright The Royal Society of Chemistry.

Because of the lower purity of the metal obtained by the extraction method, it is generally used as a front-end operation for the preparation of high-purity metals, extracting the metal to separate impurities and laying the foundation for the subsequent continuous improvement of metal purity. The essence of the extraction method is to use the extractant or adsorbent on the target metal and other impurities with a huge difference in affinity, so that the target metal and the extractant dissolved combination and impurities are not dissolved to achieve the purpose of extraction metals.

Based on the idea of extracting metals by an extraction method, researchers have carried out studies regarding the effect of different extractants on the extraction effect, in which researchers have discussed a lot about the effect of extractants. In 2020, Li et al. studied the extractant CyphosIL101. It was found to be highly thermally and chemically stable and was also less toxic than ammonium-based ILs during selective vapor extraction. Using the mechanism, CyphosIL101 has a strong affinity for indium ions and a weak affinity for impurities under HCl conditions. The extractant was found to recover indium up to 99.1%.^43^ In 2023 and 2024, Chen et al. proposed methods for stripping indium using hydrochloric acid in the presence of KI and KCl, respectively, both using crown ether solvents as extractants. It was found that B18C6 reached a 100% extraction rate in a single solution of indium in only 0.5 min, and the extraction rate of indium in a solution with impurity metal ions could reach 92.5%.^44,45^ The purification by extraction method also has a wide range of applications for the extraction of gallium, tin, germanium, lithium, and copper metals, and in 2017, Nayak et al. discussed the extraction of gallium by CyphosIL104 extractant, which can be as high as 99.8% for gallium.^46^ In 2023, El Wakil et al. studied the l5-nonyl-2-hydroxyacetophenone oxime solvent extraction of gallium and found that the protocol could achieve 99.2% extraction of gallium, and its stripping rate could reach 98.8%.^47^ In 2015, Li et al. achieved 95.5% copper extraction using the AcorgaM5640 extractant, demonstrating that the extractant can efficiently extract copper.^48^ In 2023, Wang et al. used a solvent extraction system based on tertiary amine (N235) to extract germanium, with an extraction efficiency of 95.8% at an oil phase/aqueous phase (O/A) ratio of 1/1. This idea of forming ligands in the target metal to achieve the purpose of extraction has been widely used in extraction methods.^49^ In 2024, Ni et al. used a system of TBP-FeCl_3_ to extract lithium and experimentally found that the lithium extraction rate reached 99%, and the stripping rate could reach 99.24%.^50^ In 2024, Tan et al. used YW100+D2EHPA+N235 to extract germanium, and the extraction rate of germanium by this process could reach 99.4%, and the stripping efficiency of germanium could reach 96.2%.^51^

In addition, in 2015, Li et al. investigated in detail the effect of extractant D2HEPA on indium extraction, which used D2EHPA as the indium extractant and then HCl as the indium stripping agent to strip indium from the organic phase, and the experimental efficiency of indium extraction was up to 95.4%.^48^ In 2017, Zhang et al., on the other hand, proposed a technique to recover indium from LSHD. It was found that the process could achieve a 98.18% extraction rate of indium. It is more efficient than the traditional process, the cost has been reduced, and it has great prospects in the direction of industrial application.^52^ Many extraction processes still use the solvent extraction (SX) process of traditional extraction methods, while in 2016, Nusen et al. conducted experiments using a synergistic solvent extraction (SSX) system consisting of LIX63 and Versatic10 and found that the efficiency of extracting indium could be increased up to 96% after a single contact and up to a stripping rate of 98% after 0.5 min. Its SSX process has been significantly optimized compared to the previous one, and the extraction rate of indium has been greatly improved.^53^ In 2021, the traditional process was also improved by De-la-Cruz-Moreno et al. They synthesized polymer-intercalated membranes (PIMs) by using D2HEPA as a plasticizer and extractant and found that indium leaching could be increased to 96.8% and extraction up to 94.2%. Compared to the traditional SX process, PIM is more stable and can be reused many times without the use of large amounts of organic and acidic solutions.^54^

Solid-phase extraction (SPE) is an important metal recovery technique that uses the principle that a solid adsorbent selectively captures a target constituent in an aqueous solution by adsorption. Unlike traditional liquid–liquid extraction processes, it often requires only a very small amount of organic solvent, and SPE provides a simpler and less energy-intensive separation process. In 2024, Protsak et al. discussed the effectiveness of SBA-15 and ligand-modified silica as efficient adsorbents for gallium and indium extraction. The researchers first discussed the effect of gallium ion concentration and indium ion concentration on the adsorption of these two extractants, respectively, as shown in Figure 10.^5^

**Figure 10:**
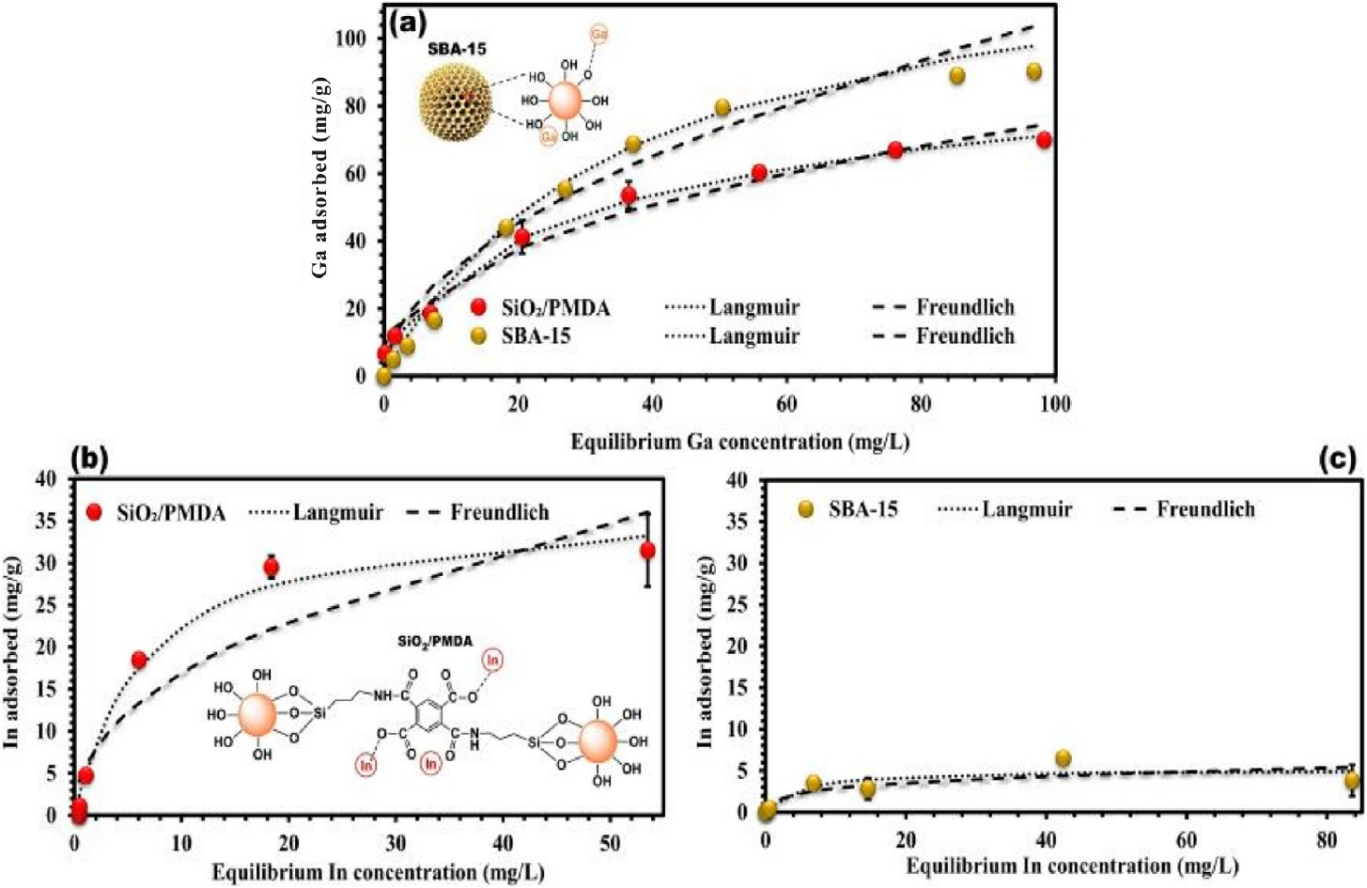
(a) Gallium adsorption isotherms for SBA-15 and SiO_2_/PMDA measured in the concentration range of 0–200 mg/L for SBA-15 and 0–150 mg/L for SiO_2_/PMDA, analyzed using Langmuir and Freundlich models. The graph includes a schematic illustration of SBA-15 with adsorbed Ga ions. (b) Indium adsorption isotherms for SiO_2_/PMDA and (c) for SBA-15 measured in the concentration range of 0–100 mg/L, analyzed using Langmuir and Freundlich models. Copyright 2024 The Author(s). Published by Elsevier B.V.

Then, they discussed the effect of adsorption time on the adsorption of the two extractants in the adsorption situation as shown in Figure 11.^5^

**Figure 11:**
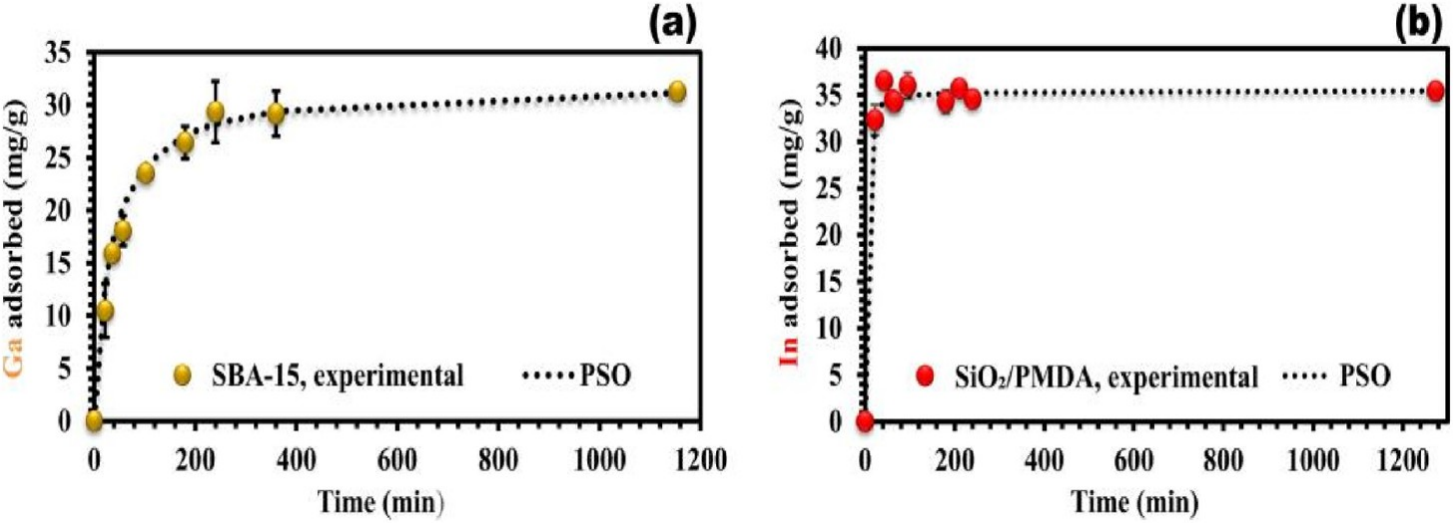
Impact of contact time on Ga adsorption by SBA-15 ((a) depicted by gold-colored points) and In adsorption by modified silica ((b) depicted by red-colored points) at initial concentrations of Ga (39 mg/L) and In (47 mg/L), analyzed with the pseudo-second-order (PSO) model and depicted as dash points. All experiments are conducted at pH 3 and 25 °C; error bars represent standard deviations from triplicate measurements. (For interpretation of the references to color in this figure legend, the reader is referred to the web version of this article.) Copyright 2024 The Author(s). Published by Elsevier B.V.

The researchers also discussed the adsorption capacity of these two extractants for different metal ions at different pH values, as shown in Figure 12.^5^

**Figure 12:**
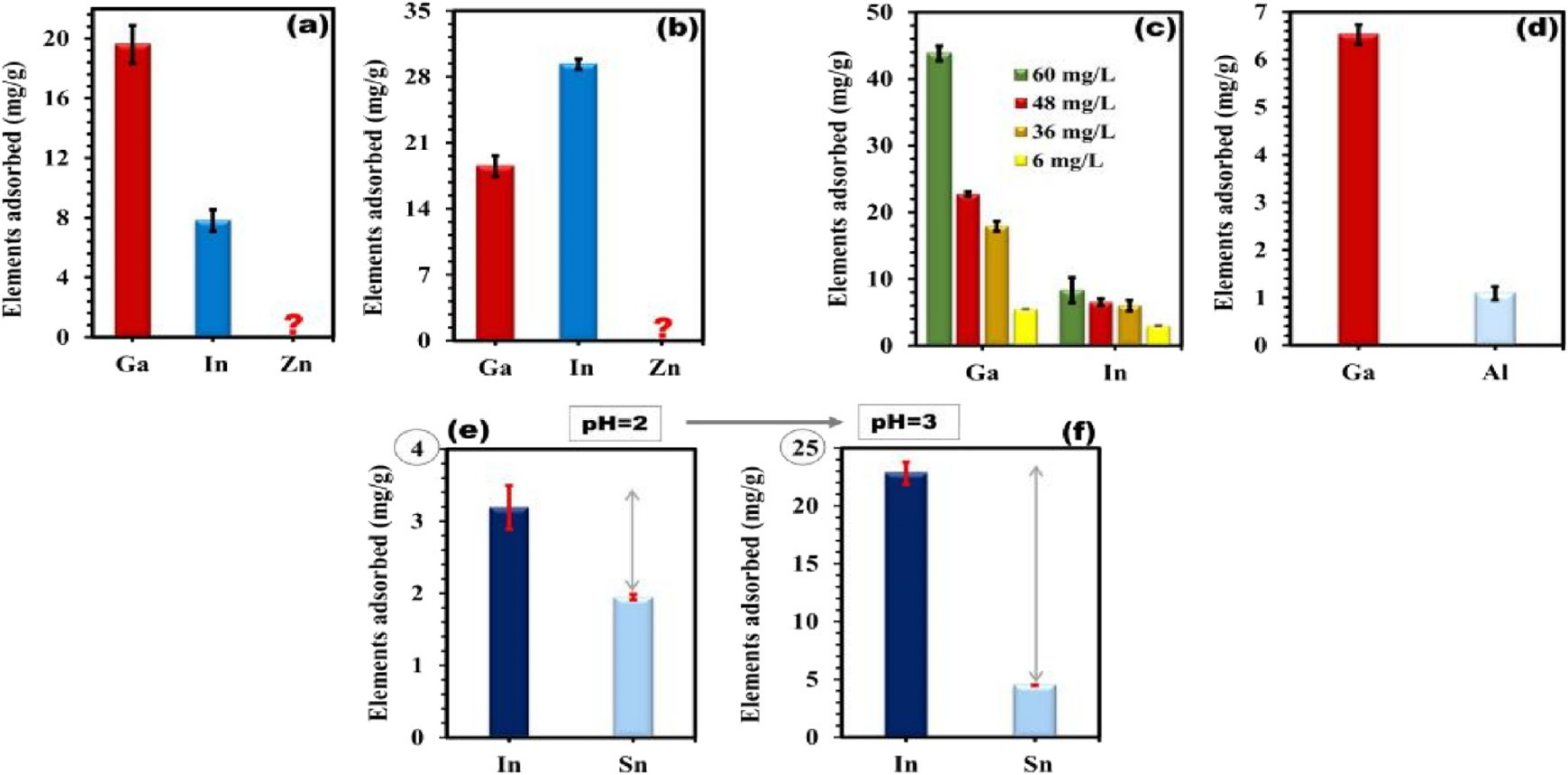
(a) Adsorption of Ga (28 mg/L), In (49 mg/L), and Zn (24 mg/L) solution by SBA-15 and by (b) SiO_2_/PMDA, with the concentrations reflecting the actual concentration ratio between these elements in In, Ga, and ZnO semiconductor target materials. The question mark signifies no affinity of the sorbents toward Zn ions. (c) Adsorption of Ga and In ions by SBA-15, with the concentration of elements indicated on the graph, and (d) adsorption of Ga and Al ions by SBA-15, with each element at a concentration of 6 mg/L. The tests were conducted at pH 3 and a temperature of 25 °C. (e, f) Adsorption of In and Sn by SiO2/PMDA at pH 2 and 3 (as indicated on the graphs) and at a temperature of 25 °C. The concentration of In in the solution was 47 mg/L, while the concentration of Sn was 4.7 mg/L, reflecting the actual concentration ratio between these elements in ITO. Error bars in all graphs represent the standard deviation of triplicate measurements. Copyright 2024 The Author(s). Published by Elsevier B.V.

They found that SBA-15 carried out the effective and selective adsorption of gallium ions, and the ligand-modified silica showed excellent performance for the extraction and isolation of indium, especially at higher concentrations. The reuse adsorption properties of SBA-15 and ligand-modified silica are observed in Figure 13.^5^

**Figure 13:**
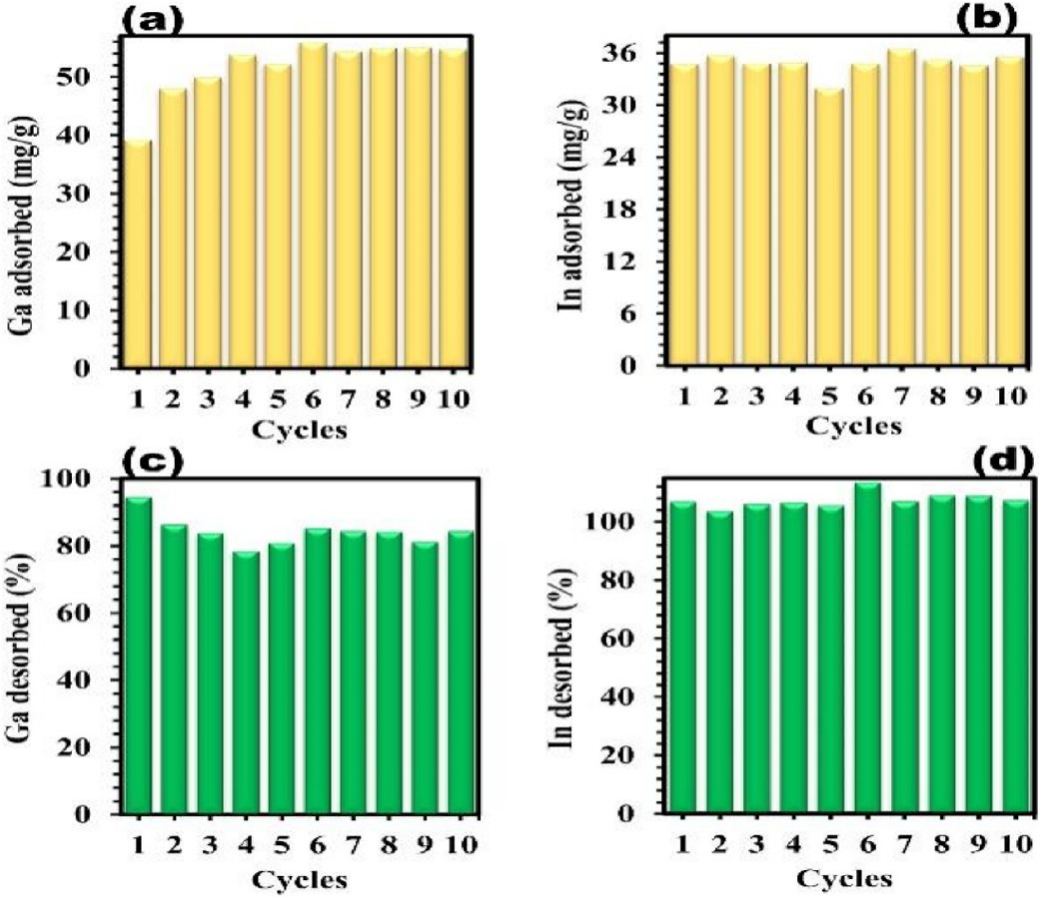
Reusability performance of SBA-15 (a) adsorption, (c) desorption with Ga ions (258 mg/L) and ligand-modified silica (b) adsorption, and (d) desorption with In ions (135 mg/L) over 10 cycles in a dynamic system. Desorption was carried out using 0.1 M HNO_3_. The conditioning solution used before each new cycle was 0.001 M HNO_3_. All tests were conducted at pH 3 and at room temperature. Copyright 2024 The Author(s). Published by Elsevier B.V.

It is found that the performance of this process is still in a stable state after many times of reuse.^5^

Researchers have found that in the pretreatment stage proper pretreatment will greatly improve the efficiency of metal extraction. In 2021, Zhang et al. proposed a method using pretreatment with NaOH combined with acid leaching. This method is optimized for the traditional acid leaching method; through the reaction between NaOH and indium, the leaching rate of indium from waste solar cells can reach 95.2%.^55^ Optimization of indium extraction was given by Yao et al. in 2023, who proposed a process of ultrasound-assisted leaching and assisted liquid film technology through high-frequency oscillation and a fuller contact to allow the indium in the ITO material of the waste liquid crystal to more fully dissolve into the leaching solution; the experiment found that this process can make the leaching rate of indium up to 97% and the final purity of indium can be up to 94%.^56^ In 2022, Wang et al. proposed the use of oxalic acid/sulfuric acid mixture leaching to extract and recover tin; the leaching rate of tin can be increased by 60%, and finally the process can recover 93.8% of tin.^57^ In 2023, Song et al. optimized the germanium extraction process using ultrasonic enhanced leaching, which allowed for a 95.19% leaching of germanium, which was 5.79% higher than the conventional leaching method.^58^

However, many processes in the pretreatment stage still have a number of environmental defects; therefore, in 2024, Jin et al. proposed a method that does not produce strong corrosive inorganic acids. Indium can be separated and recovered from ITO powders using short-chain dicarboxylic acid-ChClDESs because of their high affinity for indium and their low affinity for other impurities. Ultimately, 99.8% of the indium in the leach solution can be recovered, which is a simpler, more environmentally friendly, milder, and safer method, but this study is relatively simple to consider, so in-depth research is needed before it can be applied to the actual recovery and extraction of indium.^59^ Rafiee et al. used CH_3_COOH, an organic acid leaching electronic waste germanium extraction green method, and the program can extract more than 70% of germanium. The method CH_3_COOH has no pollution and environmental protection without environmental problems; the subsequent treatment is simple and easy to recycle and processing and on the germanium leaching facilitates the subsequent extraction and extraction of advantages.^60^

Bioleaching has the superiority of green environmental protection, high extraction efficiency, and high economic benefits, which determines its promising future. Bioleaching uses the principle that the organism utilizes its own life activities to separate metal resources from the original substance. The recovery of indium and zinc from sphalerite and flotation tailings by bioleaching and precipitation processes was proposed by Martin et al. in 2015. Leaching zinc efficiency of up to 97.7% and up to an indium content of 75.2% is observed.^61^ In 2023, Rezaei et al. also initiated a study on bioleaching to extract germanium and lithium using the waste media bioleaching process of Pseudomonas malodora and Pseudomonas coelicolor. The recoveries of germanium and lithium under optimal experimental conditions were 83 and 97%, respectively. However, biological life activities can only survive in a specific pH and the influencing factors are extremely complex; the current bioleaching method is only a relatively simple discussion; from the research to the actual application in production still needs to be developed continuously.^62^

### 2.3 Electrolysis

Electrolytic purification is one of the most commonly used techniques for the preparation and purification of high-purity metals. A high concentration of impurities containing the target metal acts as an anode, and if a direct current is passed through the electrolyte, an electrochemical reaction occurs that produces the desired high-purity metal at the cathode.^63–65^ A schematic diagram of a basic electrolysis unit is shown in Figure 14.^66^

**Figure 14:**
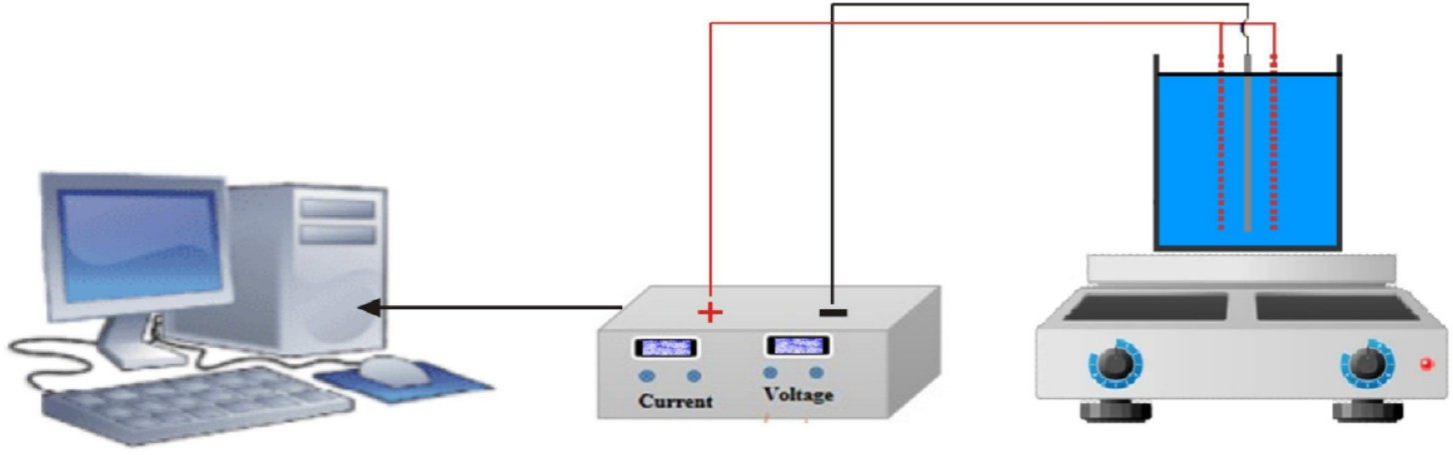
Electrolysis setup. Copyright 2024 The Authors. Published by Elsevier B.V.

The extraction-electrolysis method is also able to recover high-purity metals efficiently. In 2018, Matsumiya et al. found through research discussions and experiments that [N_1116_]­[TFSA] can be used not only as a diluent for the extractant TBP but also as an electrolytic medium for metal electrolysis, and that In­(III) can still maintain a high level of extraction in aqueous H­[TFSA] under low pH conditions and high extraction rates.^67^ This method can also be applied to metals such as Cd, Pb, Sn, etc. In 2024, Yang et al. also formed an Al–Ce alloy in LiCl–KCl molten salt for the rapid and efficient extraction of cerium,^68^ which combined molten salt electrolysis with the extraction process to realize efficient electroextraction of cerium.

In 2023, Tian et al. improved the electrolysis method by utilizing a two-stage cyclone electrowinning, and under the optimal process conditions, the purity of indium in the first stage was increased from 94.34 to 99.95%, whereas the purity of indium in the second stage was 98.95%, and the current efficiency (CE), specific energy consumption (SEC), and average cell voltage (ACV), respectively, reached 75.23%, 2.23 kW·h/kg, and 2.40 V. After a two-stage cyclone electrowinning, the combined recovery of indium was as high as 98.22%.^69^ The electrolytic process of a two-stage cyclone electrowinning is shown in Figure 15.^69^

**Figure 15:**
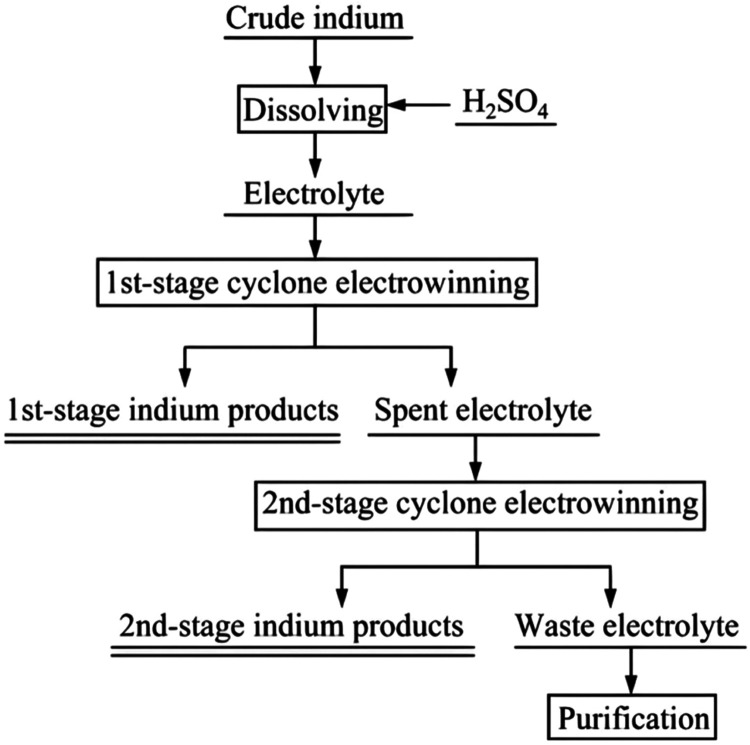
Proposed flowsheet for the purification of crude indium by a two-stage cyclone electrowinning. Copyright 2023 The Nonferrous Metals Society of China. Published by Elsevier Ltd.

Molten salt has the characteristics of high ionic conductivity, wide electrochemical window, large heat capacity, good thermal stability, low vapor pressure, etc., which is widely used in the extraction and refining and purification of metals such as aluminum, magnesium, alkali metals, rare-earth metals, refractory metals, etc.,^70,71^ and molten salt electrolysis is a method of using molten salt as an electrolyte to transform electrical energy into chemical energy for metal extraction, which is not only an innovative, economic, and green metal recovery route but also an important research direction for the development of recycling technology.^70^ A schematic diagram of a molten salt electrolysis unit is shown in Figure 16.

**Figure 16:**
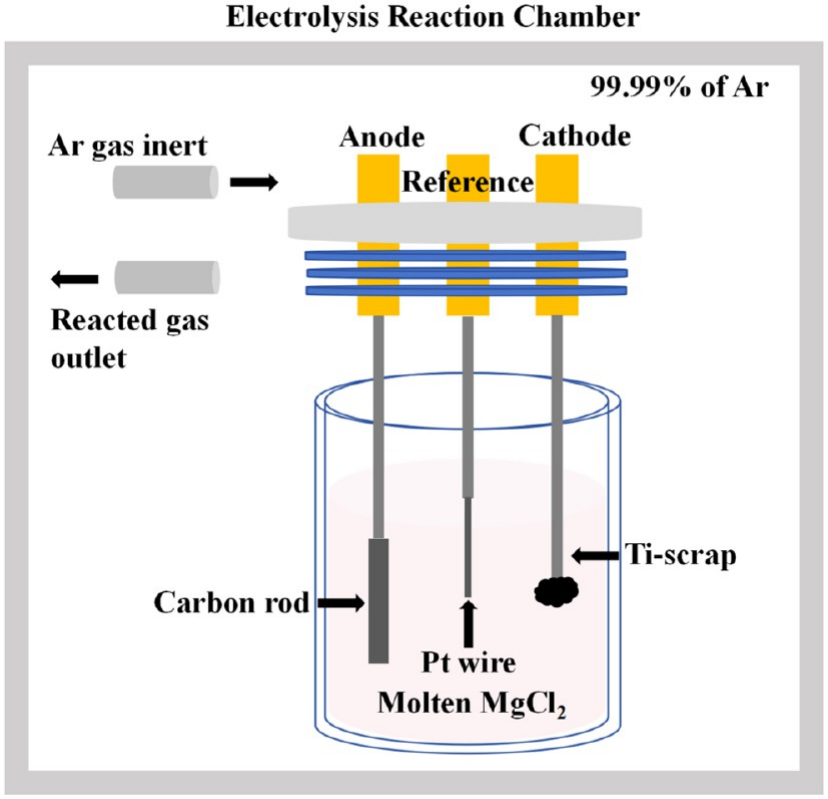
Schematic diagram of the molten salt electrolytic equipment.

In 2022, Jang et al. investigated liquid cathodic electrolysis for the recovery of cesium and strontium from LiCl–KCl eutectic salts and successfully deposited Sr on a Zn cathode with a recovery of up to 55%, whereas Cs is difficult to remove from the salt electrochemically.^72^ As shown in Figure 17,^72^ the anodic and cathodic potentials are used as a function of time during the deposition of (a) Cs and (b) Sr onto the liquid Zn cathode.

**Figure 17:**
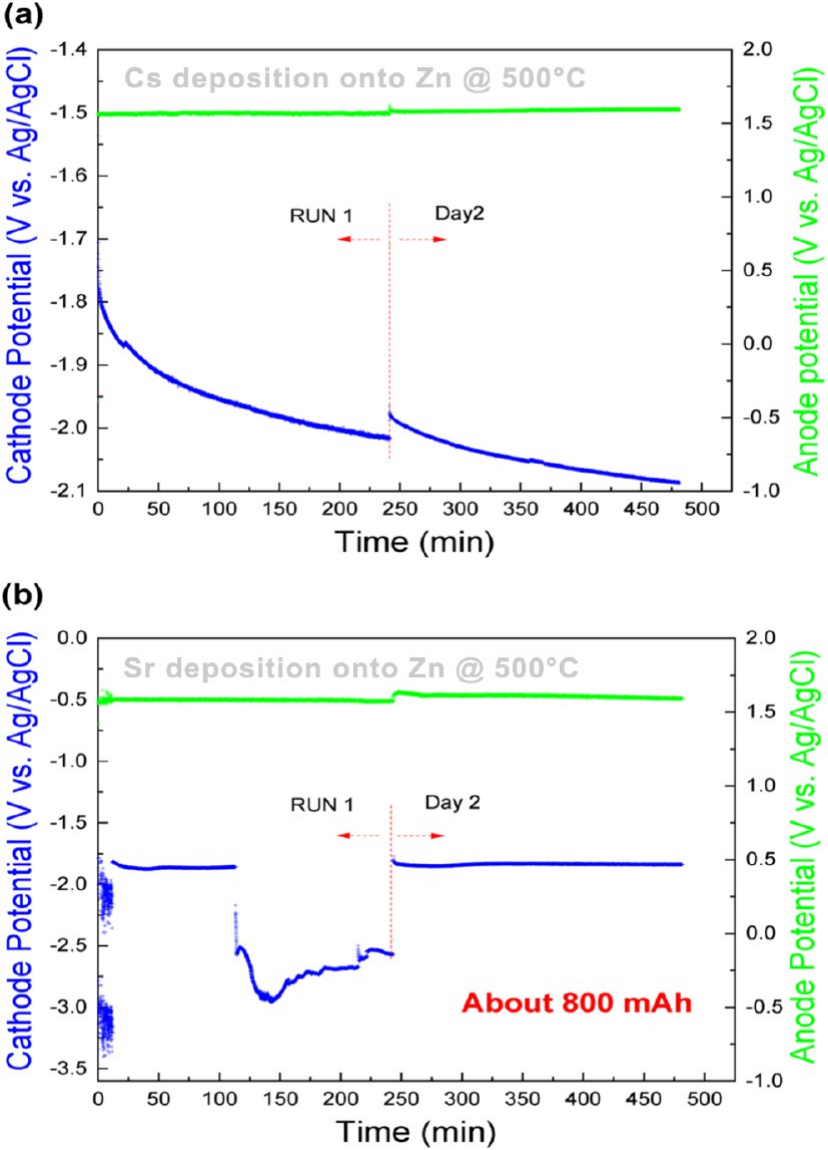
Anode and cathode potentials as a function of time during the deposition of (a) Cs and (b) Sr into the liquid Zn cathode. Copyright 2022 Korean Nuclear Society, Published by Elsevier Korea LLC.

Similarly, Cui et al. investigated the electrochemical redox process of In_2_O_3_ in molten LiCl–KCl at 450 °C as early as 2020 and successfully prepared high-purity indium under a liquid cathode.^73^ A schematic diagram of the electrolysis experimental setup is shown in Figure 18.^73^

**Figure 18:**
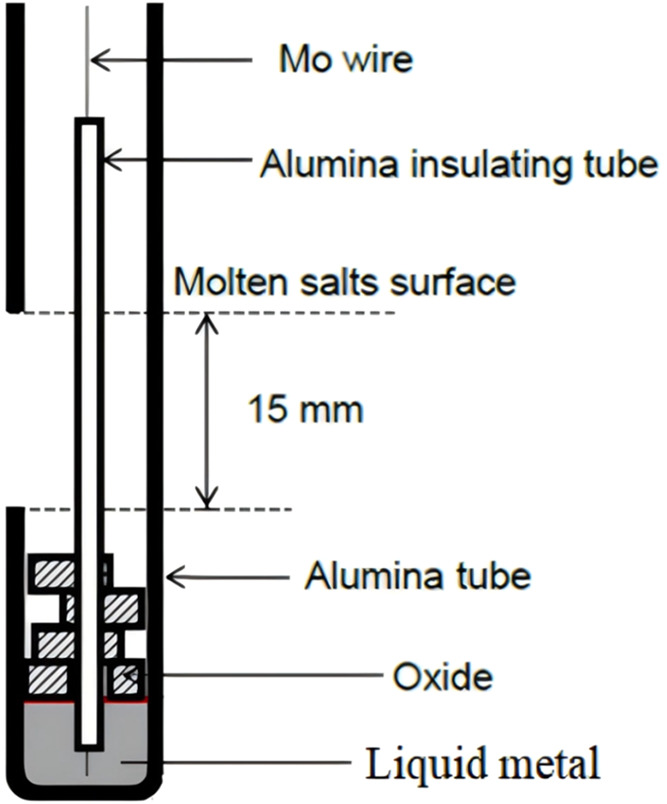
Schematic diagrams of the experimental setups for electrolysis. Copyright 2020 The Authors. Published by ESG. Published by Elsevier B.V.

In addition, in 2023, Li et al. investigated the mechanism of the soluble anode preparation of metallic iron and the purity of iron deposited at the cathode reached 99.51%.^74^ Figure 19 ^74^ shows the schematic diagram of the soluble anode preparation of metallic iron.

**Figure 19:**
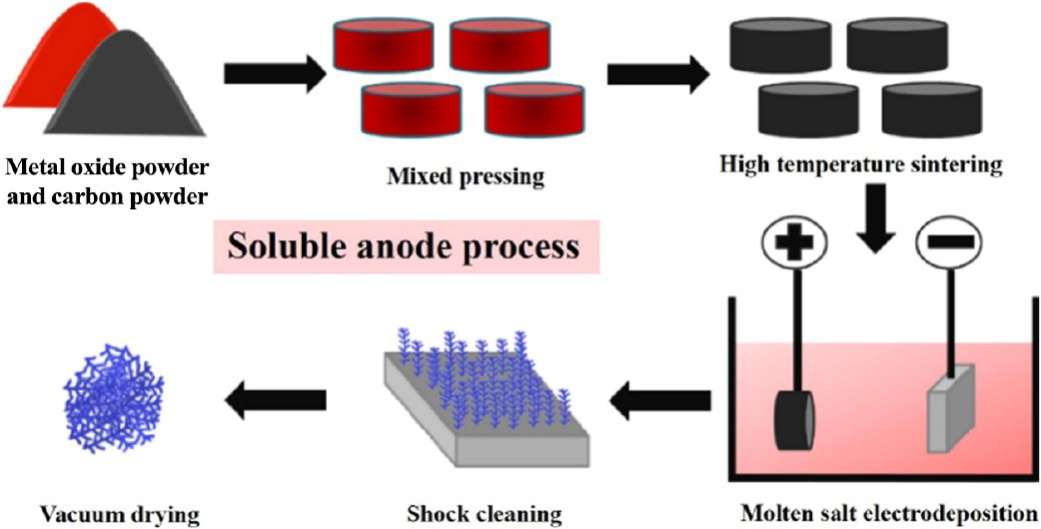
Schematic diagram of the preparation of metal iron by a soluble anode. Copyright©2023 The Authors. Published by Elsevier B.V.

In recent years, many scholars have begun to try to upgrade aluminum scrap to pure aluminum by molten salt electrolysis.^13^ In 2020, Huan et al. explored the refining of crude aluminum–silicon alloys in an AlCl_3_–NaCl–KCl molten salt system and showed that the purity of the aluminum electrodeposited on the cathode was as high as 99.3%.^75^ In contrast, Guoa et al. purified pure aluminum with 99.8% purity by electrolysis of AlCl_3_-rich molten salt using scrap aluminum as a soluble anode and a LiCl–KCl–NaCl molten salt system with a higher liquid-phase temperature and lower vapor pressure. From the point of view of purifying aluminum, molten salt electrolysis is more environmentally friendly, cheaper, and more efficient compared to polarization, disproportionation, and ionic liquid refining.^13^ And in 2019, Mohanty et al. also found that molten salt electrolysis utilizing pretreated TiO_2_ as the cathode material can produce titanium metal efficiently.^76^

Molten salt electrolysis can also be catalyzed by ultrasonic assistance. As early as 2016, Kafashan et al. utilized ultrasound in a clever combination with electrolytic purification, which greatly improved the conversion efficiency of solar cells.^77,78^ In 2019, Guo et al. also utilized ultrasound-assisted molten salt electrolysis to prepare Al–Si–Sc alloys containing the AlSi_2_Sc_2_ phase.^79,80^ Ultrasonic stirring not only accelerates the mass transfer and cleans the electrode surface but also increases the reaction rate, thus improving the electrodeposition efficiency.^81^ The principle diagram of an ultrasonic electrolysis is shown in Figure 20.

**Figure 20:**
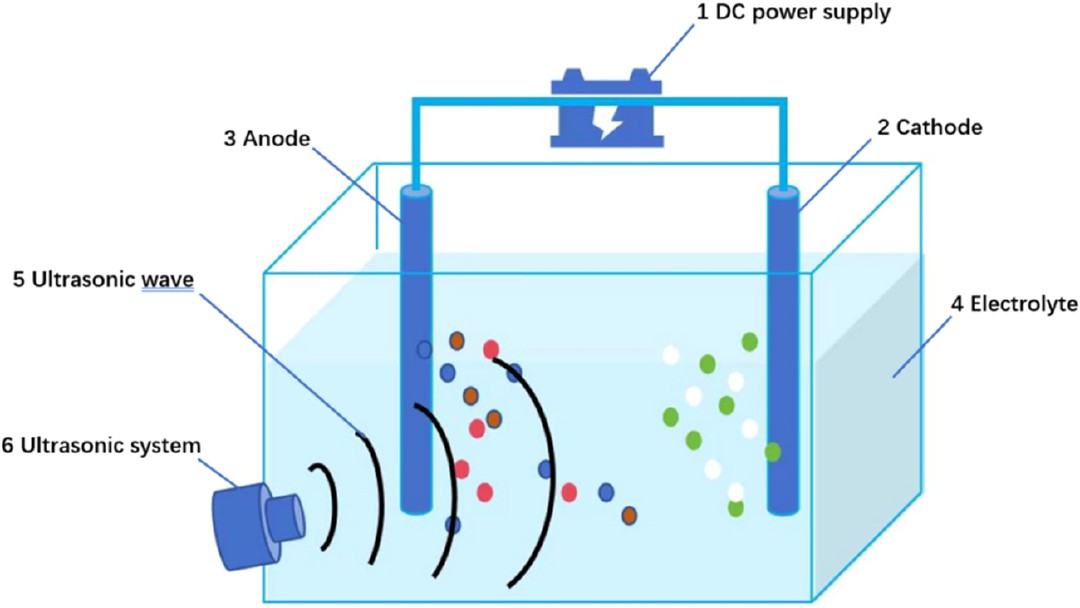
Schematic diagram of an ultrasonic electrolysis.

Not only that, in 2020, Ma et al. also investigated the use of the ultrasonic method to reduce PM produced during zinc electrolysis,^82^ which promotes the greening of the electrolysis process and provides a reference to promote cleaner production in the metal electrolysis process. Obviously, ultrasound is an excellent catalyst for the current electrolytic purification process.

In 2023, Zhu et al. innovated a powder electrolysis method to selectively extract lithium from a mixed powder of waste LiFePO_4_ and graphite,^83^ which well solved the problems of poor homogeneity of the recovered product, high energy consumption in the recovery process, and large amount of wastewater.

Membrane electrolysis for metal recovery has the advantages of a high recovery rate and high purity, while electrodialysis is a technique that utilizes ion-exchange membranes combined with a potential difference to separate substances. In 2024, Han et al. used membrane electrolysis combined with selective electrodialysis to recover iron from titanium dioxide waste acid, and the purity of the iron could be recovered to 94%,^84^ and in 2020, Pana et al. studied the direct preparation of lithium carbonate powder by membrane electrolysis for the production of lithium chloride solution.^85^ This method is expected to provide new ideas and relevant basic data for metal recycling.

### 2.4 Zone Melting

Regional refining is a deep purification of metal technology and can be used in the purification of various metals, such as germanium, tin, aluminum, cerium, indium, cadmium, gallium, etc. Its essence is to make use of the difference between the solubility of impurity elements in the solid state and the molten state of the main metal, so that the impurity precipitates or changes the distribution of impurity elements. It provides a simple and effective method for preparing high-purity metal. In theory, high-purity metals up to 8N can be obtained.^86^ When the impurity diffusion interface moves with the refining zone, the impurities at the solidification interface can move continuously in the liquid or solid as the refining zone moves. Eventually, the impurities are concentrated at the end of the ingot, while the middle part is partially purified. The solubility of most impurities in solid metals is much lower than in liquid metals, which causes these impurities to undergo strong segregation when the zone is melted. Conducive measures were used for the removal of impurities. Therefore, after several regional refinements, impurities can be substantially enriched at the tail end.^86^

In general, zone refining can be divided into a single-pass zone refining and multipass melting zone refining. Through comparison, we can see that the advantages of zone refining can be clearly seen when the multimelting point zone refining is adopted, as shown in Figure 21 ^87^ A series of closely spaced heaters are used to melt the ingot into multiple melting zones, and after multizone refining, the impurity concentration distribution reaches a steady state or limit distribution.^87^

**Figure 21:**
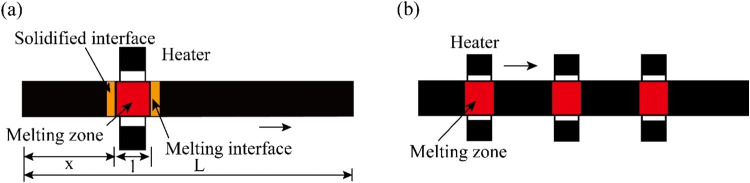
Specific process of zone refining: (a) single-pass zone refining; (b) multipass melting zone refining. Copyright 2021 by the authors. Licensee MDPI, Basel, Switzerland.

Yang et al. used the van der Waals technique to optimize the region refining process when the vacuum degree was 1 × 10^–5^ Torr or higher. The region moving speed is 7–8 cm/h; when the ratio of the ingot length to the melt zone length is 1/5–1/20, the impurity level can be significantly reduced by 10^2^–10^3^ times, from 10^13^/cm^3^–10^14^/cm^3^ of the raw material to 10^11^/cm^3^ of the zone refined ingot.^88^

At the same time, the zone melting method can also be used to extract the metal tin. In 20 years, Zhang found that the melting rate had a significant effect on the zone melting process when purifying metal tin. When the zone refining rate is reduced from 1.4 to 0.6 m/min, after 10 refinements, the metal purity in the ingot can be increased from 99.99824 to 99.99906%.^89^

In addition, in 20 years, Wan also proposed an improved zone melting method when purifying metal aluminum. As shown in Figure 22,^90^ the impurity separation effect can be greatly improved by optimizing the purification times and melting zone speed. When the melting zone velocity is 1/3 m/min, the removal rates of Fe and Cu are 99.0 and 94.75% and the removal rates of Si and Zn are 81.7 and 88.51%, respectively. After the sample was purified for 15 times at a rate of 0.5 m/min, the Al content was greater than 999994.59 pm, which met the standard of 5N high-purity aluminum.^90^

**Figure 22:**
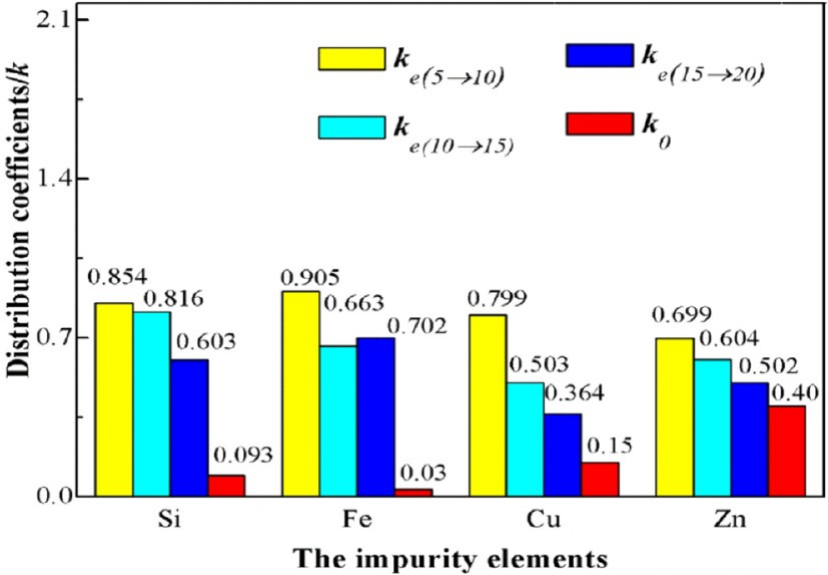
*k*_e_ of impurities at different purification times. Copyright 2020 The Author(s). Published by Elsevier B.V.

Jun purified industrial cerium by induction heating in 2017 and studied the influence of the melt zone length on the purification effect. As shown in Figure 23,^91^ with the increase of the melt zone length, the “limit distribution” curve moved upward, and the final purity decreased.^91^ Ho obtained the optimum melting zone length for 10 subregional refinements. As shown in Figure 24a, the melt zone length increases with the increase of the distribution coefficient (*k*_0_) and decreases with the increase of zone refining times. According to this step, zone refining can obtain quite a good separation effect. As shown in Figure 24b,^92^ when *k*_0_ < 1, the maximum solute removal rate decreases with the increase of the partition coefficient, and when *k*_0_ > 1, the reverse is true. The maximum solute removal rate increases with the increase of regional refining times.^92^

**Figure 23:**
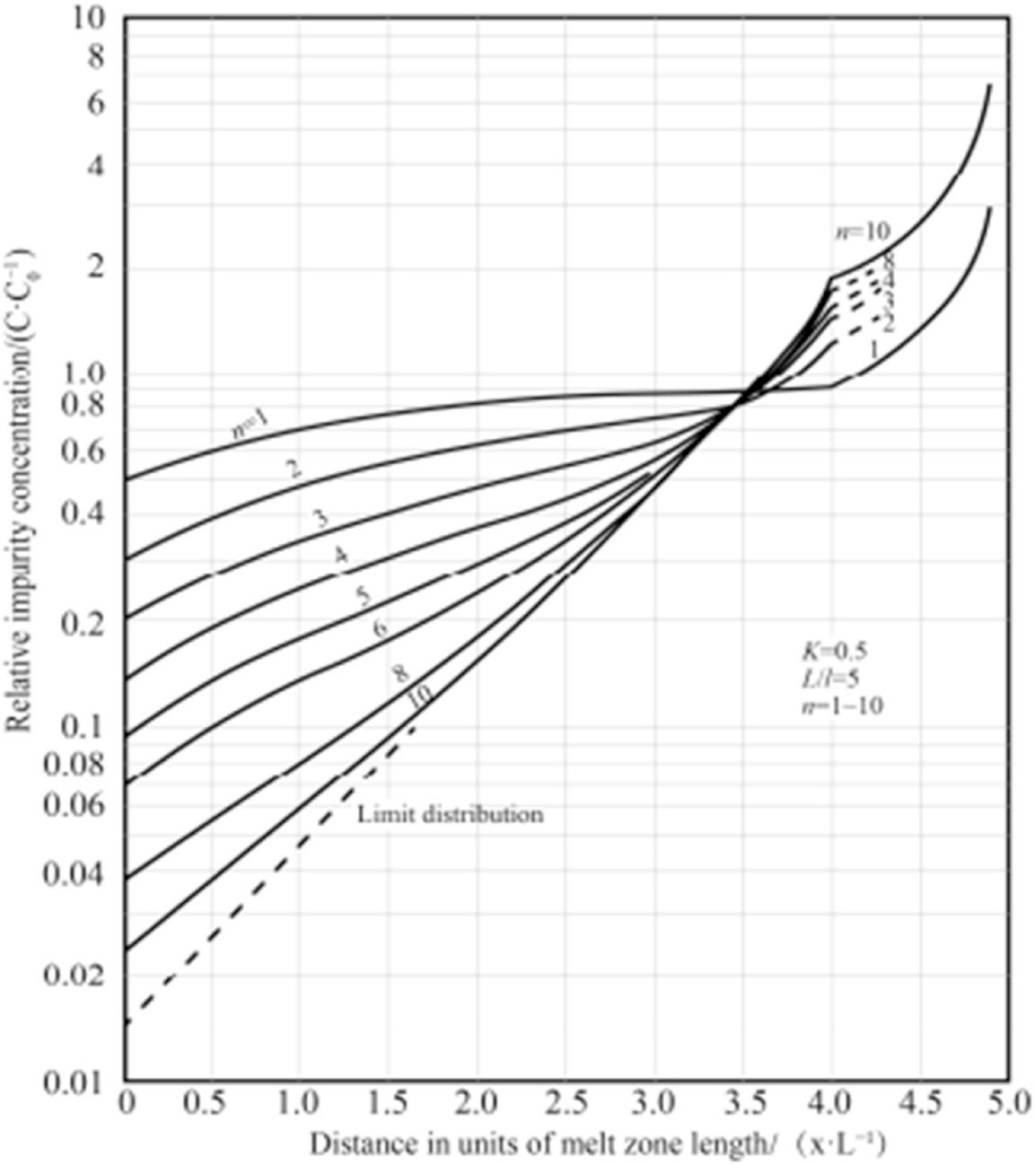
Relationship between the impurity concentration and number of zone refining passes. Copyright 2017, Northwest Institute for Nonferrous Metal Research. Published by Elsevier BV. All rights reserved.

**Figure 24:**
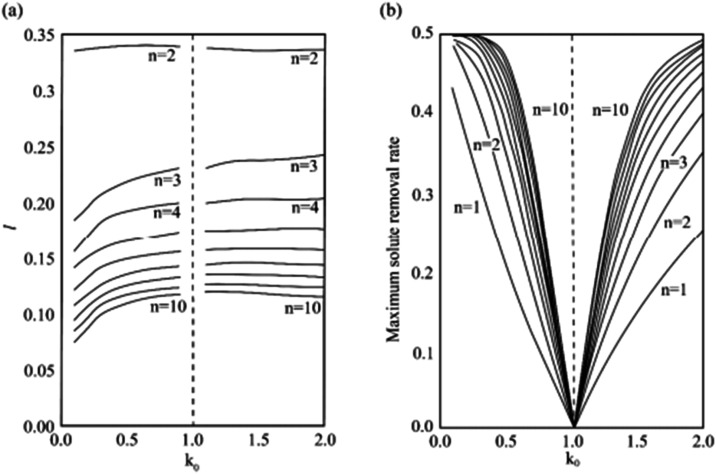
(a) Optimal zone refining length of 1–10 passes; (b) maximum solute removal rate during multipass zone refining. Copyright 1999 Elsevier Science B.V. All rights reserved.

Ghosh et al., based on the optimized parameters obtained in the study, performed 50 zone refining of 5N2 (99.9992% purity) gallium and mechanical stirring at 20–35 rpm in a directional freezing system. By glow-discharge mass spectrometry (GDMS) analysis, the purity level of refined gallium was 7N2 (99.999992%), and the total impurity concentration was reduced to 77.7 ppb relative to 24 impurities.^93^

The introduction of a current field and electromagnetic field in the purification process can drive the movement of impurities in the metal and improve the purification efficiency, so it is of wide concern. Yu et al. introduced an electric current field in the refining process, and its structure is shown in Figure 25.^87^ The segregation of impurities at the solidification interface can be improved by electromigration by applying a current field in the refining process.

**Figure 25:**
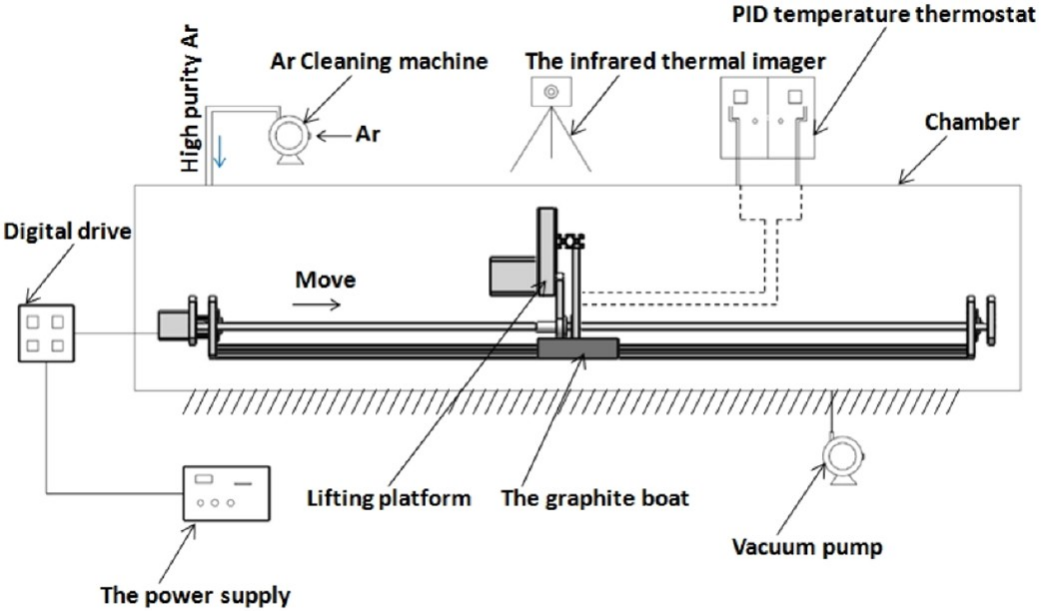
Schematic diagram of the vacuum refining-electromigration high-purity indium refining plant in the multirefining zone. Copyright 2021 by the authors. Licensee MDPI, Basel, Switzerland.

Li et al. also designed a multifurnace tube vacuum zone refining/electromigration high-purity indium refining device, as shown in Figure 26,^87^ which doubled its efficiency, and the whole process had high energy consumption and utilization rate and was environmentally friendly, and 6N indium could be produced.^87^ Dost et al. also applied a current field in the process of refining cadmium, as shown in Figure 27.^87^ The results show that the addition of external field can improve the migration rate of impurities in cadmium.^94^

**Figure 26:**
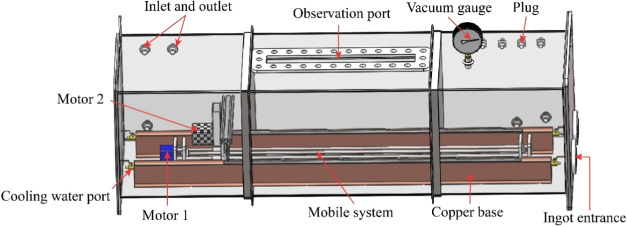
Zone refining equipment diagram. Copyright 2021 by the authors. Licensee MDPI, Basel, Switzerland.

**Figure 27:**
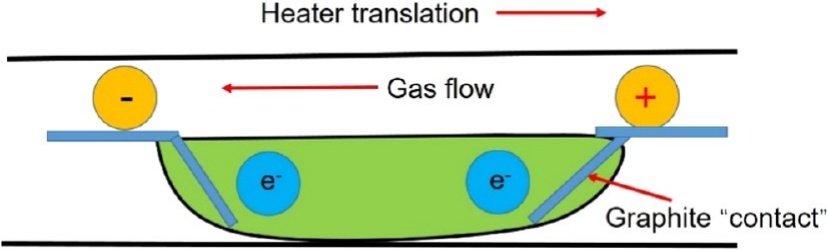
Schematic view of the ZR setup under applied electric current (electric current direction reversed in test 28). Copyright 2021 by the authors. Licensee MDPI, Basel, Switzerland.

The introduction of artificial intelligence in the field of metal purification to digitally drive the whole process of production, which can obtain a stable and simple operation process and greatly improve the production efficiency, is a future direction that must be explored. Shang et al. proposed a multiobjective optimization strategy based on machine learning to optimize the vertical zone refining process parameters of 7N grade ultrahigh purity indium. It is also concluded that when the total impurity content is 0.2–0.4 ppm, the lower velocity parameter is conducive to the removal of impurities in indium feedstock.^91^

Li et al. improved the zone melting method and developed the vertical zone refining method. Using the finite volume method, they studied the dynamic interaction between melt flow and solute distribution and successfully produced 7N indium.^95^

In view of the current market demand, the research focus of regional refining at this stage should be transferred to the combination of regional refining technology and other purification technologies to develop an ideal purification method combining electromigration/regional refining, vacuum degassing-regional refining, and other technologies to effectively remove gas impurities and obtain higher-purity materials. The second is to upgrade the regional refining equipment, improve the degree of automation, improve the regional refining process, obtain a stable and simple operation process, improve production efficiency, and reduce production costs.

### 2.5 Distillation Method

Although the above zone melting method has the advantages of wide adaptability, simple equipment operation, and high product purity, it is not suitable for impurities with a distribution coefficient close to 1.^96^ Vacuum distillation can reduce the reaction temperature and effectively reduce the influence of gas on the process, which is the most effective method for metal purification.^97^ When the same temperature is set under closed conditions, different metals have different saturated vapor pressures. According to this principle, it can be preliminarily judged whether the impurities can be separated from the base metal. In a vacuum heating environment, the impurities with saturated vapor pressure higher than the base metal enter the gas phase earlier. On the contrary, impurities with lower saturated vapor pressure remain in the melt and eventually remain in the crucible residue to achieve separation.^98^ And vacuum distillation has a wide range of adaptability and can be used in tellurium, gold, silver, magnesium, indium, praseodymium, yttrium, and a series of important metal purification experiments.

In 2021, Gao et al. used vacuum gasification-directed condensation technology to extract tellurium from lead anode slime. The experimental results showed that the volatilization rate of base metal gradually increased with the increase of the distillation temperature. As shown in Figure 28,^97^ the relationship between the temperature of each component in TLAS and the saturated vapor pressure shows that the saturated vapor pressure of tellurium, zinc, arsenic, antimony and bismuth is higher. Using this principle, the separation of tellurium was achieved.^97^ In 2024, Xu et al. used multistage distillation to improve the purity of the product. The purity of tellurium was increased from 5N to 6N.^98^ Sun and Zheng removed selenium by bubbling hydrogen in molten tellurium to form selenium hydride, which is removed with hydrogen. The raw material sample of vacuum distillation contains 8.656 × 10^–6^ (mass fraction) impurities. The total impurity content of the product after hydrogenation is 0.92 × 10^–6^ (mass fraction), which greatly reduces the content of selenium.^99^ Zhang et al. proposed a potential CD telluride photovoltaic waste recycling method in 20 years, as shown in Figure 29.^100^ The process uses a three-stage vacuum distillation, which separates from other metals by distillation or sublimation. The results show that the purity of the base metal can reach 99.97%, and the recovery rate can reach more than 99%.^100^

**Figure 28:**
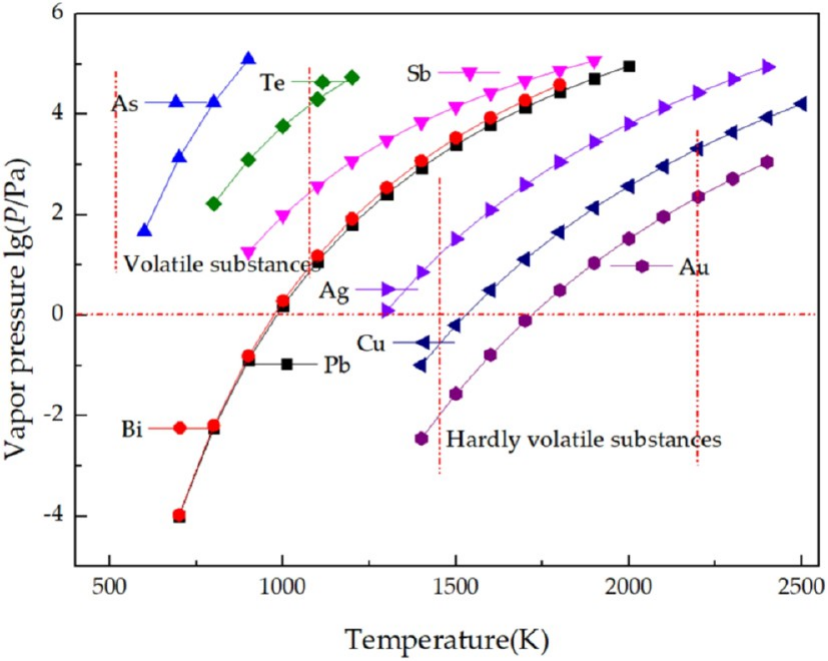
Relationship between the saturated vapor pressure and temperature of each pure substance in TLAS. Copyright 2021 by the authors. Licensee MDPI, Basel, Switzerland.

**Figure 29:**
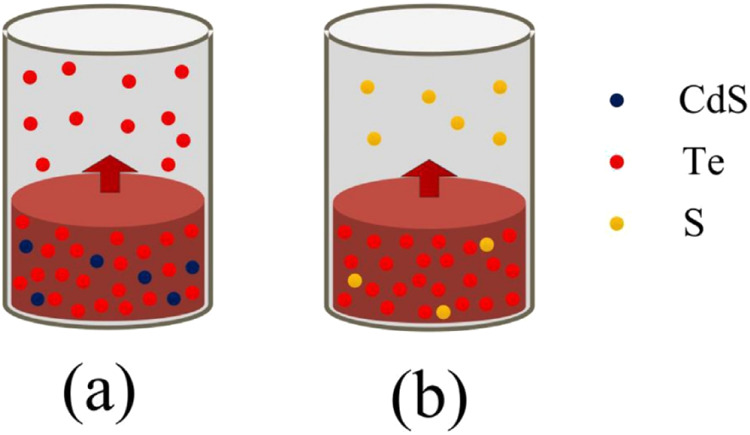
Schematic diagram of the separation of impurity elements using (a) HTVD and (b) LTVD. Copyright 2020 The Author(s). Published by Elsevier B.V.

Vacuum distillation can also be used in purifying magnesium. Liang et al. designed a horizontal hierarchical condensing vacuum furnace that has a high-precision temperature control system to control heating and condensation. This device realizes the effective separation of magnesium and impurities through the precise control of evaporation and condensation process.^101^

Similar to the introduction of current fields in the refining process of zone melting, external fields (electromagnetic and current fields) can also be used in distillation and purification. Xu et al. combined vacuum distillation with vacuum electromagnetic refining technology using electromagnetically induced electromagnetic force to suspend metal materials in quartz tubes. Under the heating and stirring of electromagnetic induction, the impurities will volatilize more fully, while avoiding the pollution of the crucible to the materials.^98^

Zhang et al. purified praseodymium and yttrium metals in a combined low- and high-temperature vacuum distillation unit, as shown in Figure 30.^102^ The purity of both metals exceeded 99.995 wt %, and the total impurity content decreased from <10202.96 to <27.45 ppmw.^102^

**Figure 30:**
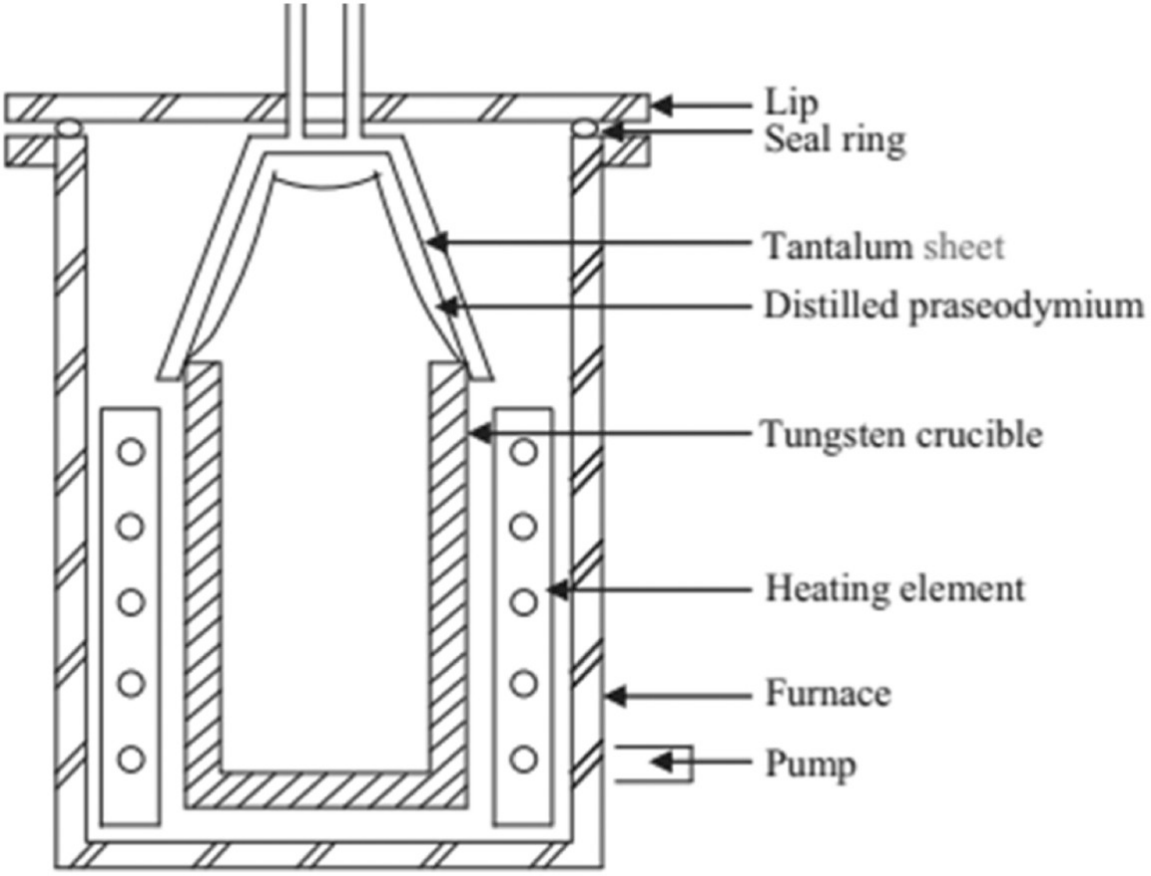
Block diagram of a pit furnace, a crucible, and a tantalum sheet assembly of a yttrium vacuum distillation system. Copyright 2014 Elsevier Ltd. All rights reserved.

The improvement of the traditional single-stage distillation unit to the multistage distillation unit has good adaptability and foresight and can greatly improve the purification rate of metals. Li et al. improved the distillation method by adopting a multistage distillation method, first low-temperature distillation and then high-temperature distillation, which greatly reduced the content of impurities. Indium was volatilized from the residue of the first stage and separated from impurity elements such as Ag, Sn, Cu, Fe, Ni, and Si with low saturated vapor pressure to produce refined indium.^103^ In 2023, Chen et al. proposed a new two-stage vacuum distillation method, using the device shown in Figure 31 ^104^ to purify crude indium into refined indium. Crude indium (99 wt %) was successfully purified to refined indium (99.995 wt %) by low-temperature distillation (1223 K, holding time 3 h) and high-temperature distillation (1473 K, holding time 5 h) at 7 × 10^–3^ Pa system pressure. According to the VLE phase diagram, the optimal low-temperature distillation temperature is 1473 K and the high-temperature distillation temperature is 1373 K, and the purification effect is good.^104^

**Figure 31:**
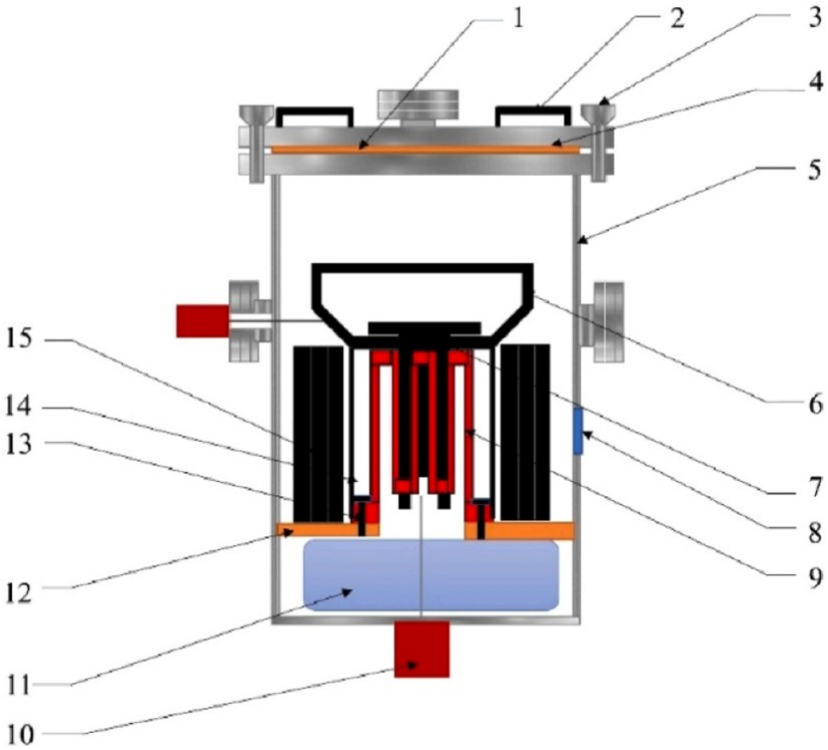
High-temperature vacuum distillation furnace: 1: sealing ring; 2: handle; 3: sealing screw; 4: furnace cover; 5: furnace shell; 6: condensation plate; 7: crucible; 8: air extraction hole; 9: heating element; 10: thermocouple; 11: insulation cotton; 12: electrode; 13: screw; 14: protective shell; and 15: graphite hard felt. Copyright 2023 The Authors. Published by Elsevier B.V.

Yi et al. also proposed an innovative and efficient oxidation-vacuum volatilization carbon reduction process for separating and enriching silver and gold from the lead anode slime. The obtained gold-rich alloy contains 67.58% Ag and 4287 g/t Au, and the recovery efficiency of Ag and Au from lead anode slime is 99.25 and 99.91%, respectively.^105^

Vacuum distillation can reduce the reaction temperature and effectively reduce the influence of gas on the process, which is the most effective method for metal purification.^97^ Compared with the traditional electrolytic process, the process of purifying indium by a two-stage vacuum distillation is short, the production cycle is short, only 3 days, and the labor cost is significantly reduced. In summary, after optimizing the refining process parameters of 7N ultrahigh purity indium in the vertical zone, it has more significant advantages than the electrolytic process.^91^ In the future, we will continue to carry out theoretical innovation and technical innovation and realize the optimization of the process of preparing high-purity metal by vacuum distillation through the combination of theoretical calculation, experimental process, and simulation. It can provide a reference for the actual production, improve production efficiency, save production costs, and achieve high-purity metal preparation.^98^

### 2.6 Other Purification Methods

In addition to the metal preparation and purification methods mentioned above, there are other methods that can effectively purify metals such as the single-crystal pulling method.

Single-crystal pulling method is to constitute the raw materials of the crystal in the crucible heating and melting, in the melt surface connected to the seed crystal pulling the melt, under controlled conditions, so that the seed crystal and the melt in the interface constantly rearrange atoms or molecules, with the cooling down of the gradual solidification and the growth of single crystals. Figure 32 ^106^ shows the straight pulling method of the device schematic diagram.

**Figure 32:**
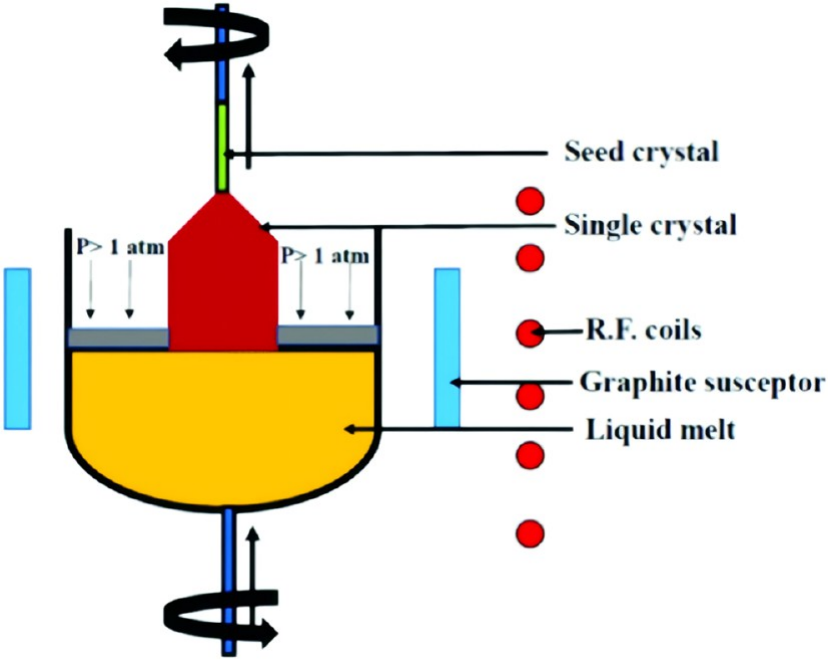
Representation of the Czochralski method. Copyright The Royal Society of Chemistry.

The control loop for the crystal diameter must be stabilized against fluctuations in growth parameters and crystal diameter.^107^ A physical model of crystal growth is shown in Figure 33.^108^

**Figure 33:**
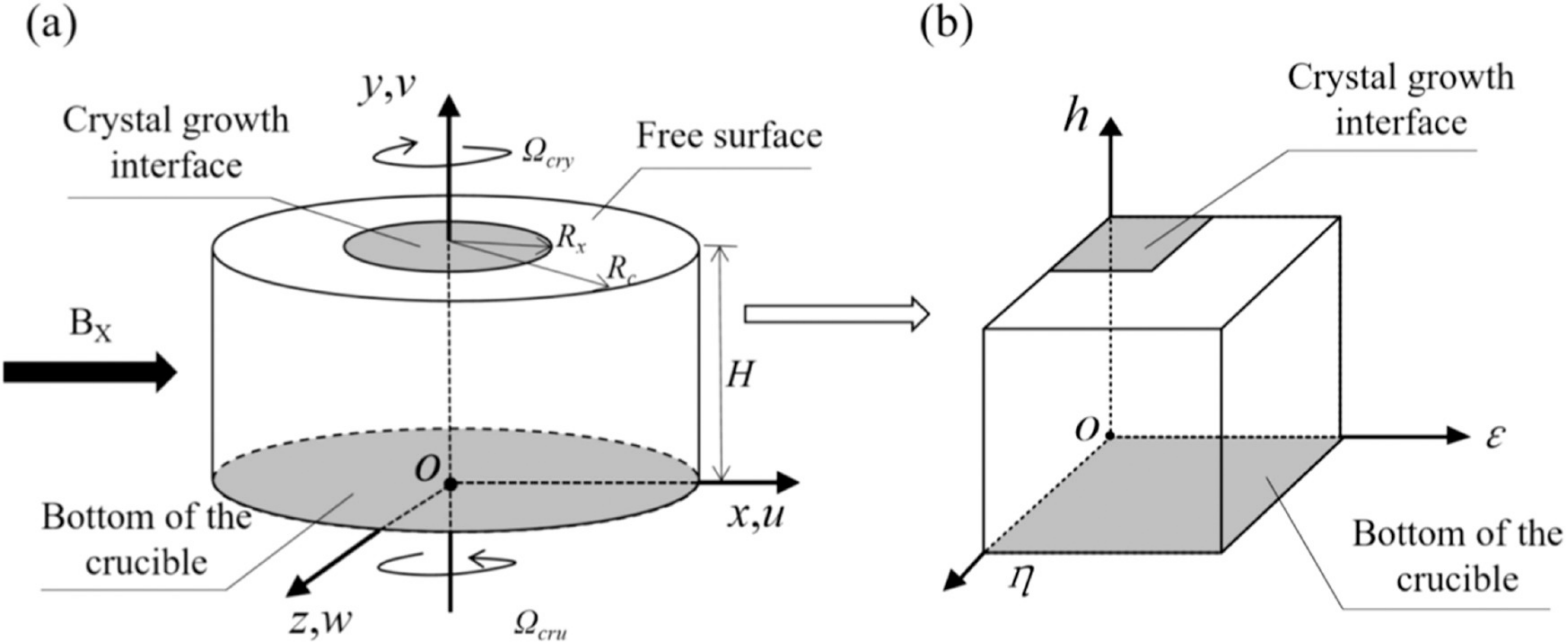
Physical model of crystal growth (a) in the Cartesian coordinate and (b) in the curvilinear coordinate. Copyright 2024 The Author(s). Published by Elsevier B.V.

In 2023, Yixuan Wang et al. found that when lanthanum was purified using single-crystal lifting and pulling, the impurity removal efficiency gradually increased with the decrease of the pulling speed; then, Al, Fe, and Ni impurities in lanthanum could be effectively removed.^109^

## 3 Summary and Outlook

With the electronic information and other high-tech fields continuing to develop and grow, the purity of high-purity metal requirements is becoming higher and higher; the current purification of high-purity metal preparation technology mainly includes extraction, ion exchange, electrolysis, regional melting, distillation, single-crystal pulling, and adsorption, and these purification techniques have their own characteristics and advantages and disadvantages, as listed in Table 2.

**Table 2:** Main Purification Preparation Methods and Their Advantages and Disadvantages

purification technology	vantage	disadvantages
extraction	the extraction method has the advantages of simpler operation, high extraction rate, fast reaction time, etc	extractants have the problems of high solubility, easy to emulsify, high cost, often use strong acid pollution, difficult to deal with
ion-exchange method	ion-exchange method has the advantages of low cost, low energy consumption, low equipment requirements, simple operation, and better separation effect	the ion-exchange method has the disadvantages of short resin life, large water consumption, and the use of strong acids that are not environmentally friendly
electrolysis	compared with the extraction method, the environmental pollution is less, the operation environment is more friendly, the process is simple, the production capacity is larger than the distillation, smelting, and other high-energy physical processes, and the equipment is simpler	consumption of electricity, some metal preparation is more difficult to control, the process produces wastewater, waste gas, waste residue, etc., the treatment cost is larger, the product is retained in the production process for a long time, the turnover speed is slow
zonal melting	with the advantages of simple and easy control, no pollution, high product purity, and wide adaptability	for impurities with distribution coefficients close to 1, the method is not applicable and has some limitations
distillation	vacuum distillation purification has a short process flow and a short production cycle, which significantly reduces labor costs	it is difficult to have a high removal rate of impurities with similar saturated vapor pressures at the same temperature, and it is necessary to take advantage of the differences in physical or chemical properties between the different impurities to be separated
monocrystalline lifting method	faster crystal growth, shorter growth cycle, easy to control the growth process, lower dislocation density	inability to produce single crystals with uniform resistivity
adsorption	with the advantages of conforming to environmental protection standards, easy to obtain, economic and other advantages, the development prospect is good	adsorbent material usage is high and costly

In summary, for the research of high-purity metal purification technology, future researchers need to focus on the following aspects.

First of all, it is necessary to address the shortcomings of the various purification technologies for high-purity metals mentioned above and continuously improve and optimize the purification technologies to narrow down the shortcomings, eliminate avoidable shortcomings, optimize the process parameters, and upgrade the purification equipment.

Second, combining the purification and preparation technologies of a variety of high-purity metals, developing an integrated purification and preparation process, effectively removing impurities, and improving the purity of metals.

Third, the practical application is the purpose of scientific research, high-purity metal purification technology, purification process, need to be production-oriented, industrialization, simplify the process, reduce costs, and continue to carry out research in the direction of low-carbon green.

Finally, we need to keep up with the changing times and actively introduce artificial intelligence. The production of high-purity metal materials is generally faced with the pain point of unstable product quality, mainly because of the long process, long cycle, and the influence of the environment, resulting in the key process not being fixed process parameters and needing to be adjusted for different actual conditions, which greatly increases the difficulty and cost of purification. And the introduction of artificial intelligence in the production construction, the use of big data and big models, through the whole process of purification and preparation, to digitally drive the process of production, improve the degree of automation, obtain a stable and simple operation process, and improve production efficiency is a future direction that must be explored.

## References

[1] Sun G., An Y., Gao S. (2024). Insights into the indium enrichment of the Ashele VMS
Cu-Zn deposit, Altay, NW China. J. Geochem.
Explor..

[2] Alfantazi A. M., Moskalyk R. R. (2003). Processing of indium: a review. Miner. Eng..

[3] Hu M., Wang Y., Chen Z., Ning S., Wei Y. (2023). Study of Indium
electrodeposition and nucleation mechanism in acidic solution using
EQCM. Electrochim. Acta.

[4] Shi C., Wang K., Chen C., Cao Y., Zhou G., Wang J., Li C. (2024). Highly selective capture
of gallium
from aqueous solutions using tetradentate amidoxime functionalized
MIL-53­(Al) nanofiber membranes. Sep. Purif.
Technol..

[5] Protsak I., Stockhausen M., Brewer A., Owton M., Hofmann T., Kleitz F. (2024). Enhanced selective extraction of indium and gallium
using mesoporous sorbents. Chem. Eng. J..

[6] Selli D., Baburin I. A., Martonak R., Leoni S. (2013). Novel metastable metallic
and semiconducting germaniums. Sci. Rep..

[7] Geng X., Liu Y., Zhang W., Wang L., Wen J., Sun J. (2022). Recent advances
in the recovery of germanium during the zinc refining process. Chem. Eng. J..

[8] Prasad S. V. S., Prasad S. B., Verma K., Mishra R. K., Kumar V., Singh S. (2022). The role and significance of Magnesium
in modern day research-A review. J. Magnesium
Alloys.

[9] Yang Y., Xiong X., Chen J., Peng X., Chen D., Pan F. (2023). Research advances of magnesium and
magnesium alloys worldwide in
2022. J. Magnesium Alloys.

[10] Balaram V., Santosh M., Satyanarayanan M., Srinivas N., Gupta H. (2024). Lithium: A
review of applications, occurrence, exploration, extraction, recycling,
analysis, and environmental impact. Geosci.
Front..

[11] Gu T., Zhang G., Wang Z., Liu L., Zhang L., Wang W., Huang Y., Dan Y., Zhao P., He Y., Zhao D. (2024). Review: The formation, characteristics, and resource
utilization of lithium slag. Constr. Build.
Mater..

[12] Liu G., Ren Y., Ma W., Morita K., Lei Y., Zhan S., Lv G., Li S., Wang Z., Li R. (2024). Recent advances and
future trend of aluminum alloy melt purification: A review. J. Mater. Res. Technol..

[13] Guo B., Wang Y., Huang Y., Peng J., Di Y., Wang C., Wang K. (2024). Upcycling of scrap aluminum to pure
aluminum through molten salt electrolysis. Process
Saf. Environ. Prot..

[14] Kumar B., Kumar P. (2022). Preparation of hybrid reinforced
aluminium metal matrix composite
by using ZrB2: A systematic review. Mater. Today:
Proc..

[15] Tang C., Deng X., Chen Y., Li Y., Deng C., Zhu Q., Liu J., Yang S. (2021). Electrochemical dissolution and recovery
of tin from printed circuit board in methane–sulfonic acid
solution. Hydrometallurgy.

[16] Guo Y., Jing J., Chen F., Wang S., Yang L. (2022). Selective
separation of tin from tin-bearing middling via sulfur roasting. Environ. Technol. Innovation.

[17] Makuei F. M., Senanayake G. (2018). Extraction
of tellurium from lead and copper bearing
feed materials and interim metallurgical products – A short
review. Miner. Eng..

[18] Zhong J., Wang G., Fan J., Li Q., Kiani M., Zhang J., Yang H., Chen J., Wang R. (2018). Optimization
of process on electrodeposition of 4N tellurium from alkaline leaching
solutions. Hydrometallurgy.

[19] Yi J., Cheng K., Zha G., Fan K., Li S., Kong X., Yang B., Liu D., Xu B. (2022). An innovative
green process for separating and enriching tellurium from lead anode
slime via vacuum gasification. J. Mater. Res.
Technol..

[20] Lv H., Zhang L., Xi X., Nie Z. (2025). Study on separation
and purification of titanium alloys (TC4–6Al-4V) by molten
salt electrolysis. Sep. Purif. Technol..

[21] Zhu F., Zhang P., Gao G., Ma Z., Mu T., Li J., Qiu K. (2024). Efficient preparation
of metallic titanium from lower
valence titanium chloride slurry by electrochemical reduction in molten
salts. J. Environ. Chem. Eng..

[22] Prasad D. S., Munirathnam N. R., Rao J. V., Prakash T. L. (2005). Purification of
tellurium up to 5N by vacuum distillation. Mater.
Lett..

[23] Prasad D. S., Munirathnam N. R., Rao J. V., Prakash T. L. (2006). Effect
of multi-pass,
zone length and translation rate on impurity segregation during zone
refining of tellurium. Mater. Lett..

[24] Niu J.-J., Wang W.-y., Li Q., Zhao C., She W.-l. (2024). Study on
ICP-MS Testing Method for High-Purity Indium Impurity Elements. Infrared.

[25] Adhikari B. B., Gurung M., Kawakita H., Ohto K. (2012). Solid phase extraction,
preconcentration and separation of indium with methylene crosslinked
calix[4]- and calix[6]­arene carboxylic acid resins. Chem. Eng. Sci..

[26] Lee S.-K., Lee U. H. (2016). Adsorption and desorption property
of iminodiacetate
resin (Lewatit TP207) for indium recovery. J.
Ind. Eng. Chem..

[27] Assefi M., Maroufi S., Nekouei R. K., Sahajwalla V. (2018). Selective
recovery of indium from scrap LCD panels using macroporous resins. J. Cleaner Prod..

[28] Li Y., Chen X., Guo B., Dai Z., Kong Z., Li F., Ou J. (2024). Synthesis of polyacrylate-divinylbenzene hydroxamic
resins and its gallium adsorption performance in sulfuric acid solution. J. Water Process Eng..

[29] Raj P., Patel M., Karamalidis A. K. (2023). Chemically modified polymeric resins
with catechol derivatives for adsorption, separation and recovery
of gallium from acidic solutions. J. Environ.
Chem. Eng..

[30] Qin Z., Wang S., Zhang S., Xie J., Zhou C.-a., Wang C., Song L., Ma K., Luo D., Yue H. (2024). Cross-linked amidoxime porous resin for selective gallium separation
in Bayer solutions: Reaction mechanism and kinetic study. Chem. Eng. J..

[31] Cruz C. A., Marie S., Arrachart G., Pellet-Rostaing S. (2018). Selective
extraction and separation of germanium by catechol based resins. Sep. Purif. Technol..

[32] He C., Qi M., Liu Y., Liu Z., Wei Y., Fujita T., Wang G., Ma S., Yang W., Gan J. (2024). Highly selective
separation of germanium from sulfuric solution using an anion exchange
D201 × 7 resin with tartaric acid. Hydrometallurgy.

[33] Arroyo F., Morillo J., Usero J., Rosado D., El Bakouri H. (2019). Lithium recovery
from desalination brines using specific ion-exchange resins. Desalination.

[34] Marinho R. S., Silva C. N., Afonso J. C., da Cunha J. W. (2011). Recovery
of platinum,
tin and indium from spent catalysts in chloride medium using strong
basic anion exchange resins. J. Hazard Mater..

[35] Alshebli R. F., Salsabila N., Yuzer B., Bicer Y. (2023). Boron and lithium recovery
from aqueous solutions by ion-exchange resin stuffed electro-electrodialysis
process with hydrogen production. J. Environ.
Chem. Eng..

[36] Min
Allah S., AlMallahi M. N., Sripadmanabhan Indira S., Al-Marzouqi A. H., Elgendi M. (2024). Recent progress in nanoparticle-based
ion exchange membranes for water desalination. Case Stud. Chem. Environ. Eng..

[37] Peng Z., Wang S., Wu Y., Liu X., Zhu M., Li P., Fu L. (2024). Novel Zr-based MOF with Ortho-hydroxyl
group selectively
traps germanium from aqueous media. Sep. Purif.
Technol..

[38] Kwak N.-S., Park H.-M., Hwang T. S. (2012). Preparation of ion-exchangeable nanobeads
using suspension polymerization and their sorption properties for
indium in aqueous solution. Chem. Eng. J..

[39] Zhu Y., Ge W., Zhang Y., Liu J., Han W., Zhang Q. (2024). Gallium extraction
from red mud via leaching with a weak acid. Process Saf. Environ. Prot..

[40] Wei S., Liu J., Zhang S., Chen X., Liu Q., Zhu L., Guo L., Liu X. (2016). Stoichiometry, isotherms and kinetics
of adsorption
of In­(III) on Cyanex 923 impregnated HZ830 resin from hydrochloric
acid solutions. Hydrometallurgy.

[41] Illés I. B., Kékesi T. (2022). The application
of selective leaching and complex anion
exchange in a novel aqueous process to produce pure indium from waste
liquid crystal display panels. J. Environ. Chem.
Eng..

[42] Pramanik S., Islam A. S. M., Ghosh I., Ghosh P. (2024). Supramolecular
chemistry
of liquid-liquid extraction. Chem. Sci..

[43] Nayak S., Devi N. (2020). Development of hydrometallurgical
process for indium recovery from
waste liquid crystal display using Cyphos IL 101. Trans. Nonferrous Met. Soc. China.

[44] Yao D., Ge T., Xu L., Chen G., Yao C., Yang C., Tian Y., Zhao Z. (2023). Complexation mechanism of crown ether
with indium in the presence of KI: Toward efficient recovery of indium
from secondary resources. Sep. Purif. Technol..

[45] Chen G., Xiong Y., Xu L., Yao C., Zhang X., Yang C., Tian Y., Zhao Z. (2024). Recovery of
indium
by solvent extraction with crown ether in the presence of KCl and
stripping with HCl: A mechanistic study. Hydrometallurgy.

[46] Nayak S., Devi N. (2017). Studies on extraction
of gallium (III) from chloride solution using
Cyphos IL 104 and its removal from photodiodes and red mud. Hydrometallurgy.

[47] El
Wakil A. F., Zaki S. A., Ismaiel D. A., Salem H. M., Orabi A. H. (2023). Extraction and separation of gallium by solvent extraction
with 5-nonyl-2-hydroxyacetophenone oxime: Fundamentals and a case
study. Hydrometallurgy.

[48] Li X., Wei C., Deng Z., Li C., Fan G., Rong H., Zhang F. (2015). Extraction and separation
of indium and copper from zinc residue
leach liquor by solvent extraction. Sep. Purif.
Technol..

[49] Wang P., Liu Z., Zhang T., Liu Z., Zhu D., Jiang T. (2023). Extraction
mechanism of germanium in sulfate solutions using a tertiary amine
(N235)-based solvent extraction system. Sep.
Purif. Technol..

[50] Ni C., Liu C., Wang J., Khan A., Zhong H., He Z. (2024). Selective
separation of lithium from the hydrochloric acid leachate of lithium
ores via the extraction system containing TBP-FeCl3. Desalination.

[51] Tan Z., Zhen Y., Wei C., Jin X., Li X., Fan G., Luo X. (2024). Organic phase modification of YW100 extraction system:
Extraction of germanium using YW100 + D2EHPA + N235. Sep. Purif. Technol..

[52] Zhang Y., Jin B., Ma B., Feng X. (2017). Separation
of indium from lead smelting
hazardous dust via leaching and solvent extraction. J. Environ. Chem. Eng..

[53] Nusen S., Chairuangsri T., Zhu Z., Cheng C. Y. (2016). Recovery of indium
and gallium from synthetic leach solution of zinc refinery residues
using synergistic solvent extraction with LIX 63 and Versatic 10 acid. Hydrometallurgy.

[54] De-la-Cruz-Moreno J.
E., Ceniceros-Gómez A. E., Morton-Bermea O., Hernández-Álvarez E. (2021). Recovery of
indium from jarosite
residues of zinc refinery by a hydrometallurgical process. Hydrometallurgy.

[55] Zhang Z., Liu M., Wang L., Chen T., Zhao L., Hu Y., Xu C. (2021). Optimization
of indium recovery from waste crystalline silicon heterojunction
solar cells by acid leaching. Sol. Energy Mater.
Sol. Cells.

[56] Huang Y.-F., Hsia W.-N., Lo S.-L. (2023). Ultrasound-assisted leaching and
supported liquid membrane extraction of waste liquid crystal displays
for indium recovery. Sustainable Chem. Pharm..

[57] Wang Y., Liu B., Sun H., Huang Y., Han G. (2022). Selective extraction
and recovery of tin from hazardous zinc-leaching residue by oxalic
acid/sulfuric acid mixture leaching and hydrolytic precipitation. J. Cleaner Prod..

[58] Song L., Zeng Y., liang M., Di H., Liu J., Yang K., Zhang L. (2023). Process optimization and mechanism
of high-efficiency germanium extracting from zinc oxide dust containing
germanium enhanced by ultrasound. Chem. Eng.
Process..

[59] Jin X., Liu G., Jin B., Rao L., Cao K., Huang Z., Chen F., Huang Q. (2024). Separation of indium and tin from
ITO powders with short-chain dicarboxylic acid-ChCl deep eutectic
solvents: Indium tin leaching and splitting mechanism. Process Saf. Environ. Prot..

[60] Rafiee P., Ghassa S., Moosakazemi F., Khosravi R., Siavoshi H. (2021). Recovery of
a critical metal from electronic wastes: Germanium extraction with
organic acid. J. Cleaner Prod..

[61] Martin M., Janneck E., Kermer R., Patzig A., Reichel S. (2015). Recovery of
indium from sphalerite ore and flotation tailings by bioleaching and
subsequent precipitation processes. Miner. Eng..

[62] Rezaei H., Shafaei S. Z., Abdollahi H., Ghassa S., Boroumand Z., Fallah Nosratabad A. (2023). Spent-medium
leaching of germanium, vanadium and lithium
from coal fly ash with biogenic carboxylic acids and comparison with
chemical leaching. Hydrometallurgy.

[63] Ciro E., Dell’Erab A., Pasqualib M., Lupia C. (2020). Indium electrowinning
study from sulfate aqueous solution using different metal cathodes. J. Environ. Chem. Eng..

[64] Wang Z., Wang Y., Liu S., Liu Y., Zhang Y., Dong Z., Cao X., Zhang Z., Liu Y. (2024). Efficient
removal of fission product thulium by electrolytic refining and high
temperature adsorption of molecular sieves to achieve the purification
and reuse of waste salt. Sep. Purif. Technol..

[65] Fan H.-Q., Li F., Zheng H.-X., Pan W.-j., Wu M.-Z., Behnamian Y., Peng J.-B., Lin D.-H. (2024). Multiple factors influencing high-purity
indium electrolytic refining. Chin. J. Chem.
Eng..

[66] Babilas D., Chromikova J., Kopyto D., Leszczyńska-Sejda K., Dydo P. (2024). Application of electrodialysis enhanced with complex formation integrated
with electrolysis for treatment of electroplating wastewaters as a
new approach to the selective copper recovery. Chem. Eng. J..

[67] Matsumiya M., Sumi M., Uchino Y., Yanagi I. (2018). Recovery of
indium
based on the combined methods of ionic liquid extraction and electrodeposition. Sep. Purif. Technol..

[68] Yang M.-C., Zhong Y.-K., Wang D.-D., Wang L., Jiang S.-L., Zhang T., Yan Y.-D., Liu Y.-L., Shi W.-Q. (2024). Rapid and
efficient extraction of cerium by forming Al-Ce alloys in LiCl-KCl
molten salts. Sep. Purif. Technol..

[69] Tian Q.-h., Dong B., Guo X.-y., Li D., Li Z.-j., Xu Z.-p. (2023). Purification of crude indium by two-stage
cyclone electrowinning. Trans. Nonferrous Met.
Soc. China.

[70] Xi X.-l., Feng M., Zhang L.-w., Nie Z.-r. (2020). Applications
of
molten salt and progress of molten salt electrolysis in secondary
metal resource recovery. Int. J. Miner., Metall.
Mater..

[71] Men X., Li S., Lv Z., He J., Song J. (2024). Kinetic analysis
of
the cathodic reduction processes in molten salt electrolysis. J. Alloys Compd..

[72] Jang J., Lee M., Kim G.-Y., Jeon S.-C. (2022). Cesium
and strontium recovery from
LiCl-KCl eutectic salt using electrolysis with liquid cathode. Nucl. Eng. Technol..

[73] Cui P., Qin B., Martinez A. M., Haarberg G. M. (2020). Electrolysis of Indium Oxide in LiCl-KCl
Based Molten Salts with a Liquid Cathode. Int.
J. Electrochem. Sci..

[74] Li H., Chen G., Liang J., Cai Z., Yang Y. (2023). Study on the
mechanism of preparing metallic iron from soluble anode. Electrochem. Commun..

[75] Huan S., Wang Y., Peng J., Di Y., Li B., Zhang L. (2020). Recovery of aluminum from waste aluminum alloy by low-temperature
molten salt electrolysis. Miner. Eng..

[76] Mohanty J., Behera P. K. (2019). Use of Pre-treated
TiO2 as Cathode Material to Produce
Ti Metal Through Molten Salt Electrolysis. Trans.
Indian Inst. Met..

[77] Kafashan H., Azizieh M., Nasiri Vatan H. (2016). Ultrasound-assisted
electrodeposition
of SnS: Effect of ultrasound waves on the physical properties of nanostructured
SnS thin films. J. Alloys Compd..

[78] Cerchier P., Dabalà M., Brunelli K. (2017). Green synthesis of copper nanoparticles
with ultrasound assistance. Green Process. Synth..

[79] Guo Z., Liu X., Xue J. (2019). Fabrication of Al-Si-Sc alloy bearing AlSi2Sc2 phase
using ultrasonically assisted molten salt electrolysis. J. Alloys Compd..

[80] Mubula Y., Yu M., Yang D., Niu H., Gu H., Qiu T., Mei G. (2024). Microwave-assisted atmospheric alkaline
leaching process and leaching
kinetics of rare earth melt electrolysis slag. Heliyon.

[81] Xiao F., Mo Z., Zhao F., Zeng B. (2008). Ultrasonic-electrodeposition
of gold–platinum
alloy nanoparticles on multi-walled carbon nanotubes – ionic
liquid composite film and their electrocatalysis towards the oxidation
of nitrite. Electrochem. Commun..

[82] Ma Z., Jiang J., Duan L., Li Z., Deng J., Li J., Zhang R., Zhou C., Xu F., Jiang L., Duan N. (2020). Ultrasonication to reduce particulate matter generated from bursting
bubbles: A case study on zinc electrolysis. J. Cleaner Prod..

[83] Zhu G., Yu D., Meugang E. F., Li H., Huan H., Guo X., Tian Q. (2023). Powder electrolysis for direct selective lithium recovery
from spent
LiFePO4 materials. Resour., Conserv. Recycl..

[84] Han F., Wang M., Liu W., Song W. (2024). Recovery of sulfuric
acid and iron from titanium dioxide waste acid by membrane electrolysis
combined with selective electrodialysis. Sep.
Purif. Technol..

[85] Pan X.-j., Dou Z.-h., Zhang T.-a., Meng D.-l., Han X.-x. (2020). Basic study
on direct preparation of lithium carbonate powders by membrane electrolysis. Hydrometallurgy.

[86] Tian Q., He Z., Xu Z., Li D., Guo X. (2024). Experimental Analysis
of High Purity Tellurium Prepared by Zone Refining. Metall. Mater. Trans. B.

[87] Yu L., Kang X., Chen L., Luo K., Jiang Y., Cao X. (2021). Research Status of High-Purity Metals
Prepared by Zone Refining. Materials.

[88] Yang G., Govani J., Mei H., Guan Y., Wang G., Huang M., Mei D. (2014). Investigation
of influential factors
on the purification of zone-refined germanium ingot. Cryst. Res. Technol..

[89] Huan Z., Zhao J., Xu J., Li Y., Pu Z., Xu B., Yang B. (2020). Preparation of High-Purity
Tin by Zone Melting. Russ. J.Non-Ferrous Met..

[90] Wan H., Zhao J., Yang B., Xu B., Duan M., Kong L., Dai Y. (2020). Study on the effective
distribution
coefficient of impurity separation in the preparation of high purity
aluminum. J. Mater. Res. Technol..

[91] Shang Z., Lian Z., Li M., Han K., Zheng H. (2023). Machine-learning-assisted
multi-objective optimization in vertical zone refining of ultra-high
purity indium. Sep. Purif. Technol..

[92] Ho C.-D., yeh H.-M., Yeh T.-L. (1999). The optimal
variation of zone lengths
in multipass zone refining processes. Sep. Purif.
Technol..

[93] Ghosh K., Mani V. N., Dhar S. (2009). Numerical
study and experimental
investigation of zone refining in ultra-high purification of gallium
and its use in the growth of GaAs epitaxial layers. J. Cryst. Growth.

[94] Dost S., Liu Y. C., Haas J., Roszmann J., Grenier S., Audet N. (2007). Effect of applied electric
current on impurity transport in zone
refining. J. Cryst. Growth.

[95] Li M., Tian Q., Wu M., Peng J., Zhang J., Chen L., Lu X., Xu Z., Zheng H. (2021). Numerical
simulation analysis on solute redistribution of In–1 wt% Sn
alloy during multipass vertical zone refining process. J. Cryst. Growth.

[96] Yuan R., LÜ C., Wan H.-l., Li S.-l., Che Y.-s., He J.-l., Song J.-x. (2022). Effect of fluoride addition on electrochemical
behaviors of V­(III) in molten LiCl–KCl. Trans. Nonferrous Met. Soc. China.

[97] Gao Z., Kong X., Yi J., Yang B., Xu B., Liu D., Wu J., Xiong H. (2021). Vacuum Gasification-Directional Condensation
for Separation of Tellurium from Lead Anode Slime. Metals.

[98] Xu Z.-p., Jia L.-l., He Z.-q., Guo X.-y., Tian Q.-h. (2024). A review
of preparing high-purity metals by vacuum distillation. Trans. Nonferrous Met. Soc. China.

[99] Sun Z.-m., Zheng Y.-j. (2011). Preparation of high
pure tellurium from raw tellurium
containing Cu and Se by chemical method. Trans.
Nonferrous Met. Soc. China.

[100] Zhang X., Liu D., Jiang W., Xu W., Deng P., Deng J., Yang B. (2020). Application of multi-stage
vacuum distillation for secondary resource recovery: potential recovery
method of cadmium telluride photovoltaic waste. J. Mater. Res. Technol..

[101] Liang D., Tian Y., Yang B., Xiong N., Wang F., Xu B. (2021). One-step preparation
of high purity
magnesium by vacuum distillation technology. Vacuum.

[102] Zhang Z., Wang Z., Miao R., Zhu Q., Chen D., Zhang X., Zhou L., Li Z., Yan S. (2014). Purification
of yttrium to 4N5+ purity. Vacuum.

[103] Li D.-s., Dai Y.-n., Yang B., Liu D.-c., Deng Y. (2013). Purification of indium by vacuum
distillation and its analysis. J. Cent. South
Univ..

[104] Chen L., Wang Y., Kong L., Xu B., Yang B. (2024). A clean and short process for the preparation of refined indium and
investigation of migration distribution pattern of impurities thallium
and tin via vacuum distillation. J. Mater. Res.
Technol..

[105] Yi J., Gao Z., Li S., San T., Kong X., Yang B., Liu D., Xu B., Jiang W. (2024). Separation
and Enrichment of Au and Ag from Lead Anode Slime by a Selective Oxidation–Vacuum
Volatilization–Carbon Reduction Process. Metals.

[106] Basu R. (2022). A review on single crystal and thin film Si–Ge alloy: growth
and applications. Mater. Adv..

[107] Uecker R. (2014). The historical
development of the Czochralski method. J. Cryst.
Growth.

[108] Zhang N., Liu D. (2024). LBM modeling of three-dimensional
mixed convection in CZ crystal growth on curvilinear coordinates system. Results Phys..

[109] Wang Y., Yu C., Zhang D., Zhang X., Li Z., Chen D., Lu W., Ke L., Li J., Han L. (2023). Exploration of impurity migration behavior in the process
of lanthanum purification by Czochralski method. Sep. Purif. Technol..

